# Mechanistic evaluation of the inhibitory effect of four SGLT-2 inhibitors on SGLT 1 and SGLT 2 using physiologically based pharmacokinetic (PBPK) modeling approaches

**DOI:** 10.3389/fphar.2023.1142003

**Published:** 2023-06-01

**Authors:** Yu Zhang, Panpan Xie, Yamei Li, Zhixing Chen, Aixin Shi

**Affiliations:** Clinical Trial Center, Beijing Hospital, National Center of Gerontology, Institute of Geriatric Medicine, Chinese Academy of Medical Sciences, Beijing, China

**Keywords:** type 2 diabetes mellitus (T2DM), SGLT2 inhibitors, physiologically-based pharmacokinetic model, inhibitory effect, sodium-glucose cotransporter 1 and 2

## Abstract

Sodium-glucose co-transporter type 2 (SGLT 2, gliflozins) inhibitors are potent orally active drugs approved for managing type 2 diabetes. SGLT 2 inhibitors exert a glucose-lowering effect by suppressing sodium-glucose co-transporters 1 and 2 in the intestinal and kidney proximal tubules. In this study, we developed a physiologically based pharmacokinetic (PBPK) model and simulated the concentrations of ertugliflozin, empagliflozin, henagliflozin, and sotagliflozin in target tissues. We used the perfusion-limited model to illustrate the disposition of SGLT 2 inhibitors *in vivo*. The modeling parameters were obtained from the references. Simulated steady-state plasma concentration-time curves of the ertugliflozin, empagliflozin, henagliflozin, and sotagliflozin are similar to the clinically observed curves. The 90% prediction interval of simulated excretion of drugs in urine captured the observed data well. Furthermore, all corresponding model-predicted pharmacokinetic parameters fell within a 2-fold prediction error. At the approved doses, we estimated the effective concentrations in intestinal and kidney proximal tubules and calculated the inhibition ratio of SGLT transporters to differentiate the relative inhibition capacities of SGLT1 and 2 in each gliflozin. According to simulation results, four SGLT 2 inhibitors can nearly completely inhibit SGLT 2 transporter at the approved dosages. Sotagliflozin exhibited the highest inhibition activity on SGLT1, followed by ertugliflozin, empagliflozin, and henagliflozin, which showed a lower SGLT 1 inhibitory effect. The PBPK model successfully simulates the specific target tissue concentration that cannot be measured directly and quantifies the relative contribution toward SGLT 1 and 2 for each gliflozin.

## 1 Introduction

Type 2 diabetes mellitus (T2DM) is a metabolic disorder characterized by hyperglycemia. Relative insulin deficiency, insulin resistance, and impaired insulin secretion are the core factors in T2DM (DeFronzo et al., 2012). Sustained hyperglycemia can lead to systemic microvascular and macrovascular changes, resulting in various complications (Meng et al., 2008). Approximately 20%–40% of patients with diabetes experience severe cardiovascular and renal complications, and one of the goals of treating T2DM is to delay the onset of such complications (Tonneijck et al., 2017). Sodium-glucose co-transporter 2 (SGLT 2, gliflozins) inhibitors were developed as oral glucose-lowering drugs that inhibit the sodium-glucose co-transporter 2 in the renal proximal tubules. The mechanism of action of SGLT 2 inhibitors is independent of endogenous glucose production (Demin et al., 2014) and insulin regulatory pathways (Scheen, 2015 **)**. Extensive cardiovascular and renal outcome studies have demonstrated that SGLT2 inhibitors are effective in reducing the risk of composite major adverse cardiovascular events (MACE) or attenuating the progression of chronic kidney disease (CKD) in type 2 diabetes, which is better than other glucose-lowing medications. Also, SGLT2 inhibitors were superior to glucagon-like peptide-1 (GLP-1) receptor agonists in reducing hospitalization for heart failure (HHF) and a composite kidney outcome (Davies et al., 2022). Therefore, the 2019 American Diabetes Association (ADA) guidelines strongly recommend that SGLT2 inhibitors should be added to their prescriptions for T2DM patients with CKD or heart failure (Buse et al., 2020).

**FIGURE 1:**
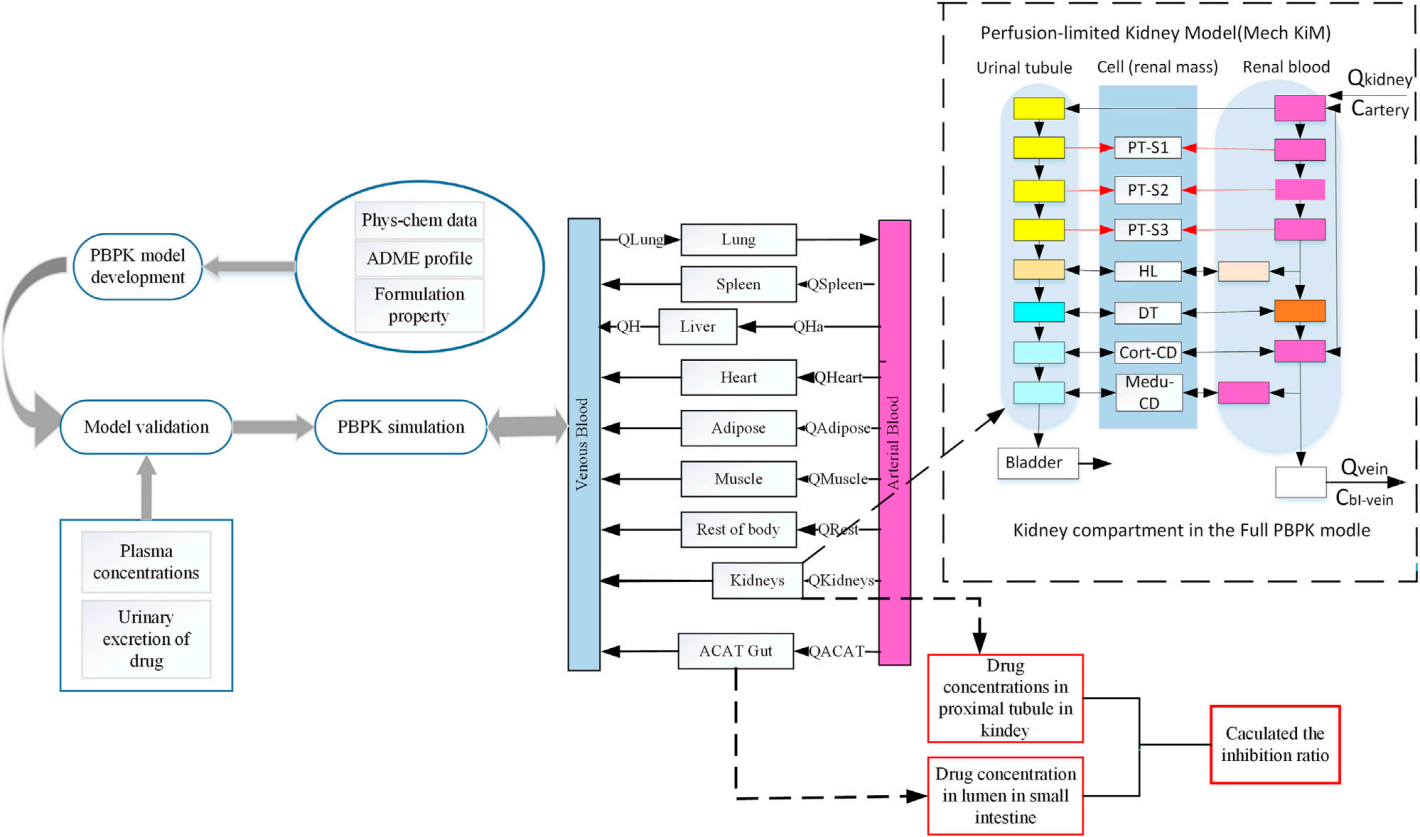
Schematic representation of workflow of this study and a basic framework of the developed SGLT 2 inhibitors PBPK model. This schematic diagram shows the mechanistic perfusion-limited models contained in this study (Emami Riedmaier et al., 2016). The perfusion-limited models were added in this extended SGLT 2 inhibitor model used in this study to describe glucose filtration, reabsorption, and excretion. SGLT 2 is expressed in the S1 and S2 segments of the proximal tubule. SGLT 1 is expressed in the S3 segment of the proximal tubule. Phys-chem, physicochemical; Q, Plasma flows rate to tissue; QH, Blood flows in the hepatic vein; QHa, Blood flows in the hepatic artery; ACAT, Advanced compartmental absorption and transit; PT, Proximal tubule; HL, Henle’s loop; DT, Distal tubule; Me-du-CD, Medullary collecting duct.

SGLT 2 is a low-affinity, high-volume transporter protein in the epithelial cells within the proximal tubule (especially in the S1,2 segment - Figure 1) and promotes 80%–90% of renal glucose reabsorption from the glomerular filtrate (Chen et al., 2015). SGLT 1 is a high-affinity, low-capacity transporter of the proximal straight tubule (especially in the S3 segment - Figure 1) responsible for reabsorbing the remaining filtered glucose. In addition, SGLT 1 is an essential glucose transporter responsible for dietary glucose absorption in the apical membrane of the small intestine epithelium. Mori et al. Therefore, the role of SGLT1 in both kidney and intestinal glucose reabsorption makes this transporter a potential therapeutic target for diabetes. Some studies confirmed the feasibility of developing therapeutic agents that only inhibit SGLT1 (Song et al., 2016). Therefore, the different inhibitory effects on SGLT1 may cause differences in efficacy and safety among SGLT2 inhibitors. A better understanding of SGLT inhibition activities may provide opportunities for modulation development to minimize the occurrence of adverse reactions. Also, quantitatively differentiating the capacity of SGLT inhibition is helpful in increasing the benefit to risk ratio of this treatment medication.

**FIGURE 2:**
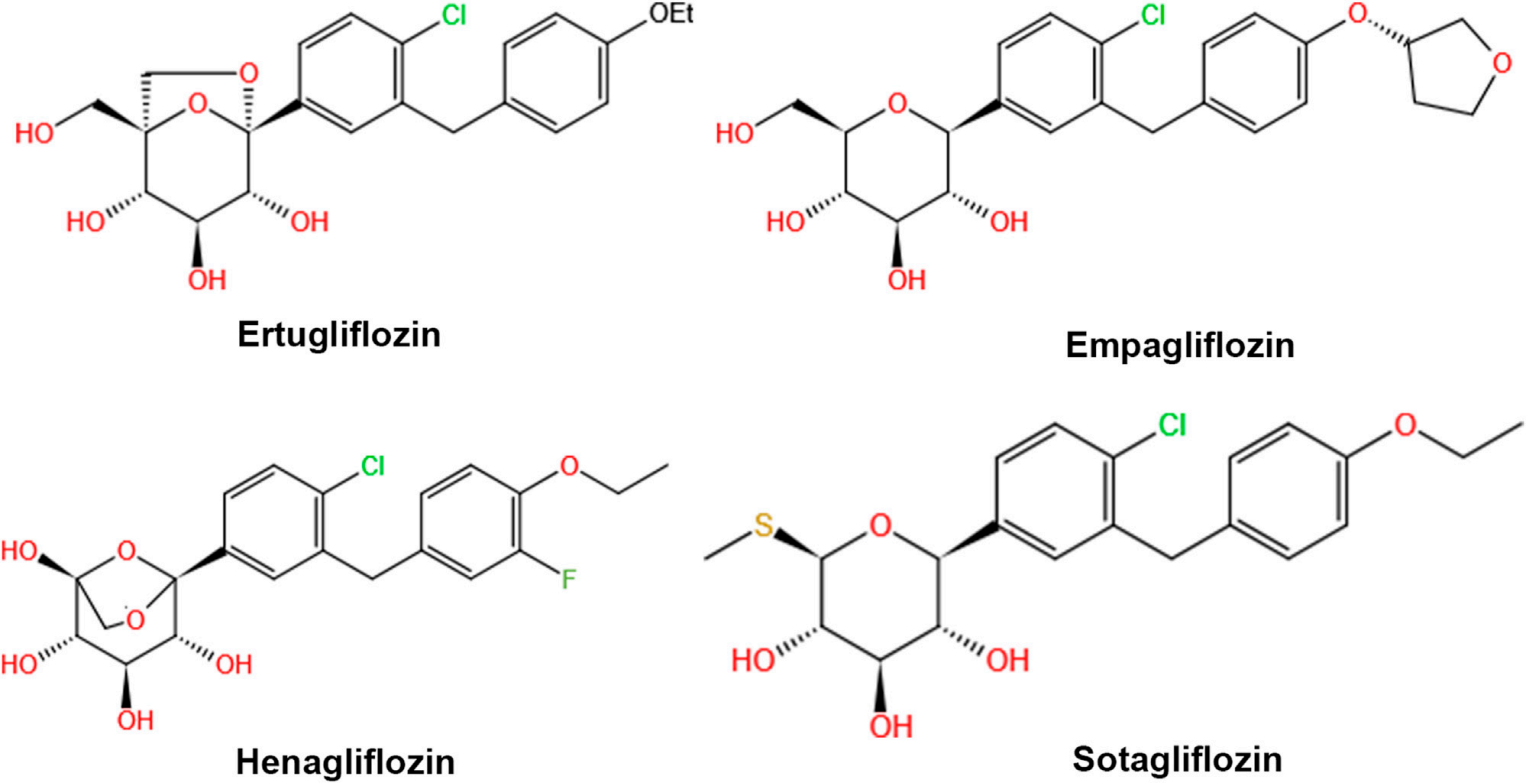
Chemical structures of ertugliflozin, empagliflozin, henagliflozin, and sotagliflozin.

The chemical structures of all four SGLT 2 inhibitors are shown in Figure 2. Empagliflozin and ertugliflozin have received regulatory approval in the US and EU for the treatment of T2DM in adult patients. Compared to canagliflozin, empagliflozin and ertugliflozin have the highest affinity for SGLT 2 *versus* SGLT 1 (>2000-fold) (Garcia-Ropero et al., 2018). The approved dose of ertugliflozin is 5 mg once daily, with a maximum of 1 mg per day. The recommended dose of empagliflozin is 10 mg once daily, which can be titrated up to 25 mg per day. Henagliflozin is a novel SGLT 2 inhibitor, which has a similar structure to ertugliflozin (Wang et al., 2016), and both 5 mg and 10 mg are the doses authorized for clinical use in china. Henagliflozin has an 1818-fold higher SGLT 2 selectivity as compared to SGLT transporters (Yan et al., 2014). Sotagliflozin was approved for treating both T1DM and T2DM in the EU, and 200 mg or 400 mg are the doses authorized for clinical use (He et al., 2022). It is the first dual SGLT 1/SGLT 2 inhibitor to receive regulatory approval in the EU. Sotagliflozin has a slightly higher affinity towards SGLT 2 transporters than SGLT 1 (only 20-fold), and it is more potent than other gliflozins in inhibiting SGLT 1 (Garcia-Ropero et al., 2018).

Because the intestinal and renal drug concentrations affect the hypoglycemic effects of SGLT 2 inhibitors, target tissue concentrations are particularly essential to address pharmacological questions. By integrating system-property and drug-property parameters, a bottom-up PBPK models allow mechanistic extrapolation from *in vitro* and *in silico* studies and increase the understanding of drug pharmacokinetic and disposition characteristics (Sager et al., 2015). PBPK modeling is now widely used as it is significant for predicting concentration-time profiles of specific tissues that are difficult to obtain from the clinical trial (Moss and Siccardi, 2014). It could better describe the relationship between drug efficacy and target tissue exposure (Kong et al., 2020). This study aimed to develop a PBPK model that quantifies the distribution kinetics of SGLT 2 inhibitors in the intestine and kidney proximal tubules. Finally, we combined with the given equation to compare the relative inhibitory capacity of SGLT 1 and 2 transporters in ertugliflozin, empagliflozin, henagliflozin, and sotagliflozin under physiological conditions. Our study provides a mechanistic understanding of the pharmacokinetic characteristics of SGLT 2 inhibitors. Also, the results of our study not only contribute to understanding the pharmacological mechanism and support the feasibility of future development in the SGLT 2 inhibitor class and the growing field of personalized medicine.

## 2 Materials and methods

### 2.1 Data used for model establishment

Data were collected from clinical trials and *in vitro* studies to estimate SGLT 2 inhibitor concentrations in intestinal segments and the proximal tubules of the kidney. A summary of data applied for the model establishment is shown in Table 1.

**TABLE 1:** Parameters required for PBPK model establishment.

Parameter type	Ertugliflozin Fediuk et al. (2020); Callegari et al. (2021)	Empagliflozin Sarashina et al. (2013); Chen et al. (2015); Yakovleva et al. (2019)	Henagliflozin Yan et al. (2014); Zhang et al. (2021)	Sotagliflozin Cefalo et al. (2019)
Molecular weight (g·mol-1)	436.8	450.9	454.1	424.9
logP	2.5	2.31^a^	2.72^a^	4.09^b^
Solubility (mg·l-1)	0.163^a^	0.13^a^	0.182	0.0136^a^
Peff (×10-4 cm·s-1)	4.1^b^	1.08^b^	6.7^b^	1.72^a^
Fup (%)	6.4	13.8	5	2.7
Rbp	0.66	0.66	0.55^b^	0.6
Total clearance (L·h-1 kg-1)	0.14	0.15	0.11	0.35
Total hepatic clearance (L·h-1 kg-1)	0.13^b^	0.09^b^	0.09^b^	0.33^b^
Renal clearance (L·h-1 kg-1)	GFR×Fup	GFR×Fup	GFR×Fup	GFR×Fup
${ki}_{SGLT1}$ (nM)	1888.66	797.61	4184.52	7.02
${ki}_{SGLT2}$ (nM)	0.88	0.64	2.38	1.41

^a^ ADMET, PredictorTM;

^b^ Optimized. Rbp, blood-to-plasma concentration ratio; logP, octanol/water partition coefficient; Peff, effective permeability; f_up_, fraction unbound in plasma; GFR, glomerular filtration rate; K_i_, Inhibitory constant; K_m_, MichaelisMenten constant; V_max_, maximum rate of metabolism formation.

#### 2.1.1 Clinical pharmacokinetic data

We collected the available clinical data from phase I trials treated with gliflozin. With the help of resources (PubMed, ClinicalTricals.gov, DrugBank), we obtained important clinical data from four clinical studies, which contained the clinical data of gliflozins in a variety of doses, such as empagliflozin (1–100 mg) (Sarashina et al., 2013), ertugliflozin (1–25 mg) (Li et al., 2020; Li et al., 2021) henagliflozin (2.5–200 mg) (Zhang et al., 2021), and sotagliflozin (200–400 mg) (He et al., 2022). The databases were used for PBPK model construction and prediction accuracy qualification, respectively. At the same time, the mean plasma glucose, mean creatinine clearance rate, urine excretion of unchanged drugs, and treatment regimen and dosages were collected.

#### 2.1.2 Physicochemixal properties

The physicochemical properties parameters of gliflozins for the development of PBPK models are obtained from the references (Sokolov et al., 2020; Callegari et al., 2021; Zhang et al., 2021), for sotagliflozin (Cefalo et al., 2019), published and internal Sanofi data were used. Other parameters were predicted and optimized by the module of ADMET Predicted and Optimization of GastroPlus^TM^. The observed mean plasma concentrations of gliflozins over time were obtained from references and digitized with GetData Graph Digitizer V2.26.

### 2.2 Software

The PBPK model was developed utilizing GastroPlus^TM^ (version 9.8.5, Simulations Plus, Inc., Lancaster, CA, United States). The plasma concentration-time curves were digitized from the published clinical studies using GetData Graph Digitizer (Version 2.25.0.3 2, S. Fedorov) software. Visualizations of model simulations were performed with the Origin package version 2022.

### 2.3 PBPK model development

This model was described using 14 tissue compartments connected by arterial and venous blood flows (Figure 1). The advanced compartmental absorption and transit (ACAT) model (Yu and Amidon, 1999), and perfusion-limited mechanistic kidney model are included to explain the absorption, distribution, metabolism, and excretion (ADME) characteristics after oral administration. The ACAT model defined the gastrointestinal was consistent with nine compartments. In the ACAT model, the duodenum, jejunum, and renal tissue compartments are the targeted tissues where gliflozin expresses its inhibitory effects on SGLT transports.

The extended model depicts the physiological structure of the kidney in this study (Figure 1). Almost all of the glucose is filtered by the kidney and enters the proximal tubule, and filtered glucose can distribute along the proximal tubules. Then all excess filtered glucose will accumulate in the bladder and excrete in urine. SGLT transporters are predominantly expressed in proximal tubules, where it mediates glucose reabsorption (Bailey, 2011). In our renal model, we divided the proximal tubules into three compartments. In the S1 and S2 segments of the proximal tubules, most of the filtered glucose is reabsorption by SGLT 2 transporter. SGLT1 is expressed in the S3 segment of the proximal tubule, where SGLT 1 transports the remaining glucose into cells (Yakovleva et al., 2019).

Some physicochemical properties and biopharmaceutical parameters, such as octanol/water partition coefficient (log P) and solubility, were obtained from the ADMET predictor model or Drugbank database. To characterize the absorption process of gliflozins in the ACAT model, we choose the opt lgD SA/V version 6.1 to predict the absorption scale factors (ASF) model in each gastrointestinal compartment. Furthermore, we defined the tissue plasma partition coefficients (Kp values) of gliflozins by visually fitting the distribution phase and found that the Lukacoval method (Rodgers-single) could better calculate the Kp values. Compared to other methods, the Kp values predicted by the Lukacova method calculated the steady-state volume (Vss) values were closer to the observed data. The lukacoval method was used to explain the distribution state between drug-binding components based on physicochemical, fraction unbound, and LogP (Rodgers et al., 2005; Rodgers and Rowland, 2006), plasma protein binding, and blood-to-plasma ratio. The above parameters are presented in Table 1. The liver and kidney were assumed to be the only elimination tissues, and the plasma clearance of healthy subjects was calculated using the PK plus model. The mass of unchanged drug in the urine was relatively low (Chen et al., 2015; Li et al., 2021; Zhang et al., 2021), indicating that the renal mostly depended on filtration clearance. The renal clearance was predicted by the glomerular filtration rate (GFR) × fraction of drug unbound in plasma (fup), and the predicted renal clearance (CL_R_) was nearly consistent with the published data.

### 2.4 PBPK model simulation

Based on the pharmacokinetic parameters of various dosages following single-dose administration in healthy volunteers, the PBPK model was developed, optimized, and validated.

#### 2.4.1 Ertugliflozin

The ertugliflozin PBPK model was used to develop and validate using oral plasma concentration-time data in healthy subjects from various ethnic subgroups (Dawra et al., 2019; Li et al., 2021). We developed an original PBPK model by combining *in vitro* and clinical data after a single dose of 1 mg. After developing the original PBPK model, simulations were performed at doses of 5 mg, 15 mg, and 25 mg. By comparing plasma concentration-time profiles and pharmacokinetic parameters, we again verified the developed PBPK model.

#### 2.4.2 Empagliflozin

The raw databases of empagliflozin consisted of forty-eight Japanese subjects (Sarashina et al., 2013), who respectively received single doses of 1 mg, 10 mg, 25 mg, and 100 mg. The model was developed using oral plasma pharmacokinetic data of 1 mg and 10 mg, and other mean clinical pharmacokinetic data were used to validate the PBPK model.

#### 2.4.3 Henagliflozin

Clinical pharmacokinetic data for oral administration of henagliflozin in healthy subjects were obtained from the references (Zhang et al., 2021). The data of six healthy adults were used to develop the model, who were respectively assigned to receive single doses of 2.5 mg, 5 mg, 10 mg, 25 mg, 50 mg, 100 mg, and 200 mg. Among, mean plasma concentration data after oral 2.5 mg and 5 mg were used to develop and optimize the model. The pharmacokinetic data after a single dose of 10 mg, 25 mg, 50 mg, 100 mg, and 200 mg were used to verify the model.

#### 2.4.4 Sotagliflozin

To establish a reliable PBPK model, a clinical study was obtained from 24 Chinese treated with 200 mg and 400 mg, respectively (He et al., 2022). The pharmacokinetic data were used to develop the PBPK model, and the prediction accuracy of the optimized sotagliflozin PBPK model was further validated using the mean plasma concentration data from multiple-dose of 200 mg and 400 mg.

### 2.5 PBPK model qualification

Two decisions were carried out to evaluate the accuracy and reliability of PBPK models. The first was to assess the fit of the plasma concentration-time curve graphically predicted using non-compartmental from GastroPlus 9.8.5 and the observed profiles from clinical trials. Additionally, the observed and predicted values of the main pharmacokinetic variables were compared, such as C_max_ (maximum plasma concentration), AUC (area under the concentration-time curve at steady state), and T_max_ (time corresponding to C_max_), and the fold error between the observed and predicted values of different doses were used to evaluate the accuracy of the established PBPK model (FE; Eq. 1; (2). The fold errors of pharmacokinetic parameters are within two-fold (Parrott et al., 2005; Jones et al., 2006; De Buck et al., 2007), suggesting that the established model can successfully predict the ADME of gliflozins in healthy subjects.

$$Fold-error=observed\;/\;predicted\;\left(observed\;value\;>\;predicted\;value\right)$$ (1)

$$Fold-error=predicted\;/\;observed\;\left(observed\;value\;<\;predicted\;value\right)$$ (2)

### 2.6 Renal model validate

To perform accurate simulations of gliflozin concentrations in the kidney, based on the demographics published in the corresponding clinical study protocols, a virtual population of 100 individuals was developed to verify the mechanistic kidney model following the clinical approval dose. The virtual subjects were used in 100 simulations to predict the 90% confidence interval (90% CI) of gliflozin cumulative urinary excretion. The NHANES database in Gastroplus was utilized for the development of virtual populations. In the created virtual populations, the percentages of males, age, height, BMI, and weight were consistent with the clinical trials published in the references. Population-dependent human physiological characteristics, such as tissue volumes and blood flow rates, were automatically produced based on input from the subjects’ demographics.

### 2.7 Calculation of inhibition rate

The glucose reabsorption/absorption rate:

$$V_{0}=\frac{V_{\max}\times \left[S\right]}{K_{m}+\left[S\right]}$$ (3)

Where V0 is glucose reabsorption or absorption rate, Vmax is the maximal rate of reabsorption or absorption by SGLT-mediated (Yakovleva et al., 2019), and Km refers to the glucose affinity constants of the SGLT transporter, which were obtained from the references (Lu et al., 2014). S is the glucose concentration at the target tissue, and the glucose level of the intestinal lumen was defined as 1 670 mmol**·**L^-1^ (Mori et al., 2016). The glucose concentration filtered into the glomerulus was assumed to be equal to the plasma glucose concentration and was used for reabsorption, and the flow rate of glucose into S3 was expressed by Egfr (Samukawa et al., 2017).

The inhibition effect of SGLT-mediated glucose absorption/reabsorption is characteristic:

$$V_{i}=\frac{V_{\max}\times \left[S\right]}{K_{m}\times \left(1+\frac{\left[I\right]}{K_{i}}\right)+\left[S\right]}$$ (4)

Where I denote SGLT inhibitor concentration in the target tissue, and Ki is the inhibitory constant.

The affinity of SGLT inhibitor to SGLT transport:

$$K_{i}=\frac{{IC}_{50}}{1+\frac{\left[S^{\prime }\right]}{{K_{m}}^{\prime }}}$$ (5)

Where S′ is the substrate concentration for determining IC50 values (Mascitti et al., 2011; Grempler et al., 2012; Yan et al., 2014; Cefalo et al., 2019; Wang et al., 2019; Fediuk et al., 2020). Km’ is the Michaelis constant of SGLT.

The total inhibitory rate of SGLT was expressed:

$$Inhibition\;ratio=\left(1-\frac{V_{i}}{V_{0}}\right)\times 100$$ (6)

## 3 Result

### 3.1 Simulation of the oral pharmacokinetic profile and the optimization of the PBPK model

#### 3.1.1 Ertugliflozin

The PBPK model for ertugliflozin was developed based on the published physicochemical parameters (Callegari et al., 2021). According to the representation in the method section, we simulated the ADME process of ertugliflozin 1 mg. The simulated plasma concentration-time profile fits well with the observed. The pharmacokinetic data for a single dose of 5 mg, 15 mg, and 25 mg in different populations were used to qualify the accuracy of the model further. Table 2 displays that the fold error values were within the 2-fold range.

**TABLE 2:** Observed and simulated pharmacokinetic parameters after oral administration in healthy subjects.

Dose (mg)	AUC (ng·h·mL-1)				C_max_ (ng·mL-1)				Tmax h)		
	Predicted	Observed	Fold error	Predicted		Observed	Fold error	Predicted		Observed	Fold error
Ertugliflozin											
15^a^	1238.5	1291.7	1.04	263.08		266.17	1.02	1		1	1
1^b^	75.4	68.09	1.11	18.82		17.9	1.05	1		1	1
5^b^	393.82	433.79	1.1	95.5		79.7	1.19	1.04		1.02	1.02
25^b^	2485.6	2151.4	1.16	437.61		432	1.01	1.16		1.25	1.07
Empagliflozin											
1	141.73	129.01	1.1	16.19		14.63	1.11	1.25		1.25	1
10	1493.5	1286.5	1.16	147.55		148.83	1.01	1.3		1.5	1.15
25	2919.5	2843.7	1.03	317.5		284.5	1.12	1.78		2	1.12
100	9507.7	10590	1.11	1201.4		1192.1	1.01	2.12		2.5	1.18
Henagliflozin											
2.5	395.1	408	1.03	52.1		54.8	1.05	0.96		1.5	1.56
5	766.09	796.69	1.04	106.92		117.37	1.09	0.96		1.5	1.56
10	1413.4	1415.6	1	205.1		161.7	1.26	1.16		1.75	1.51
25	3642	3384.1	1.08	457.07		406	1.12	1.1		2	1.82
50	7449.5	7671.8	1.03	964.07		941.7	1.02	1.1		1.5	1.36
100	13930	13440	1.04	1732.5		1460	1.19	1.6		2	1.25
200	28630	28480	1.01	3305.1		3140.5	1.05	1.68		1.5	1.12
Sotagliflozin											
200	475.36	631.14	1.33	115.82		90.9	1.26	0.72		1	1.39
400	928.08	1129.8	1.22	195.54		151	1.29	0.72		1	1.39

^a^ Non-Asian;

^b^ Asian;

#### 3.1.2 Empagliflozin

**FIGURE 3:**
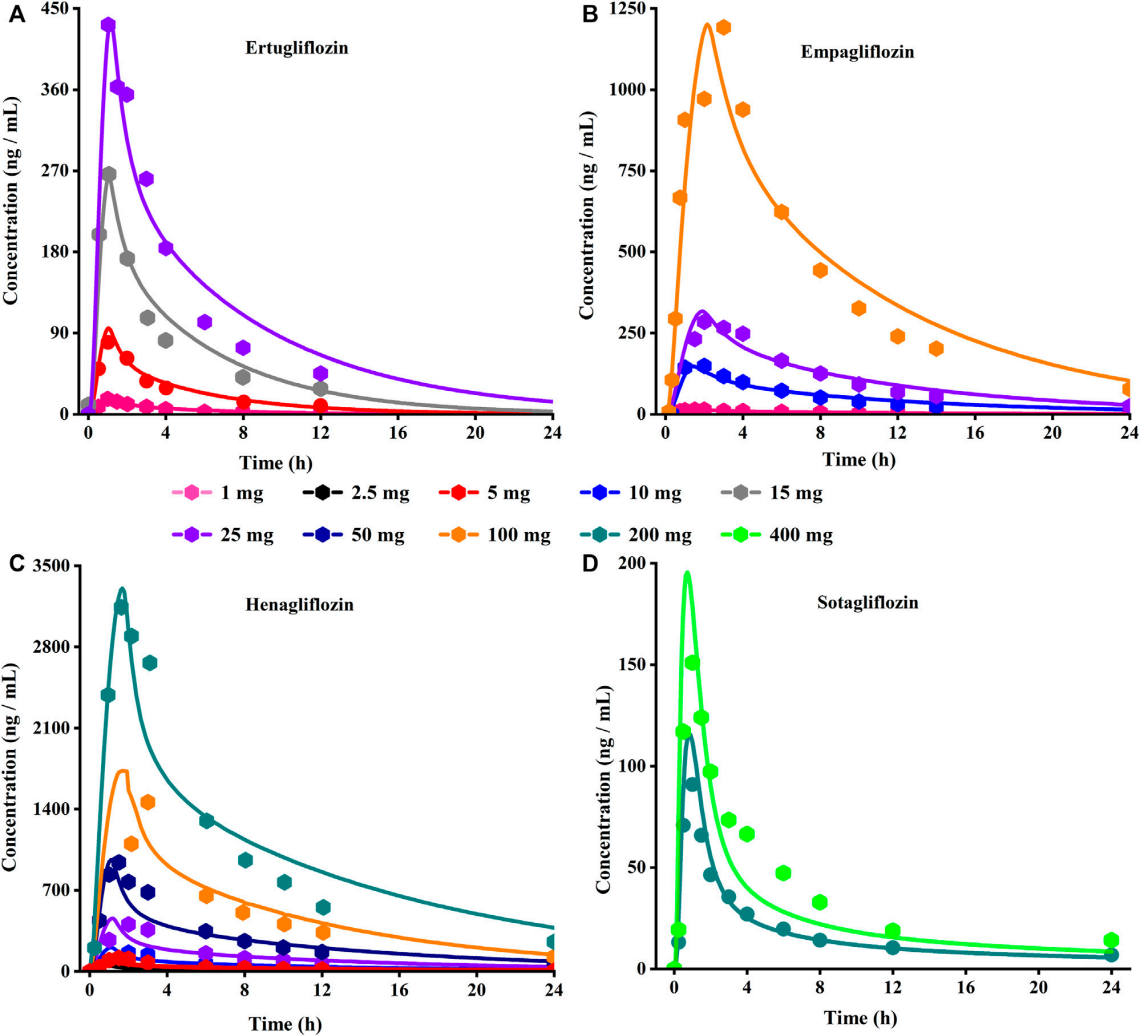
Predicted (lines) and observed (symbol) plasma concentration-time profiles after a single dose with the updated PBPK model. **(A)** Ertuglifozin**; (B)** Empagliflozin; **(C)** Henagliflozin; **(D)** Sotagliflozin. PBPK, Physiologically based pharmacokinetic.

Using the parameters from references and the ADMET Predictor module, we simulated plasma-concentration profiles of empagliflozin 1 mg. The simulated oral plasma concentration-time curves corresponded well with the observed. The predicted and observed pharmacokinetic parameters and corresponding fold error were summarized in Table 2. The baseline PBPK model was validated by simulating the plasma concentration-time profiles of 10 mg, 25 mg, and 100 mg, which accurately matched the observed profiles (Figure 3). The predicted pharmacokinetic parameters were also in good agreement with the observed values.

#### 3.1.3 Henagliflozin

**FIGURE 4:**
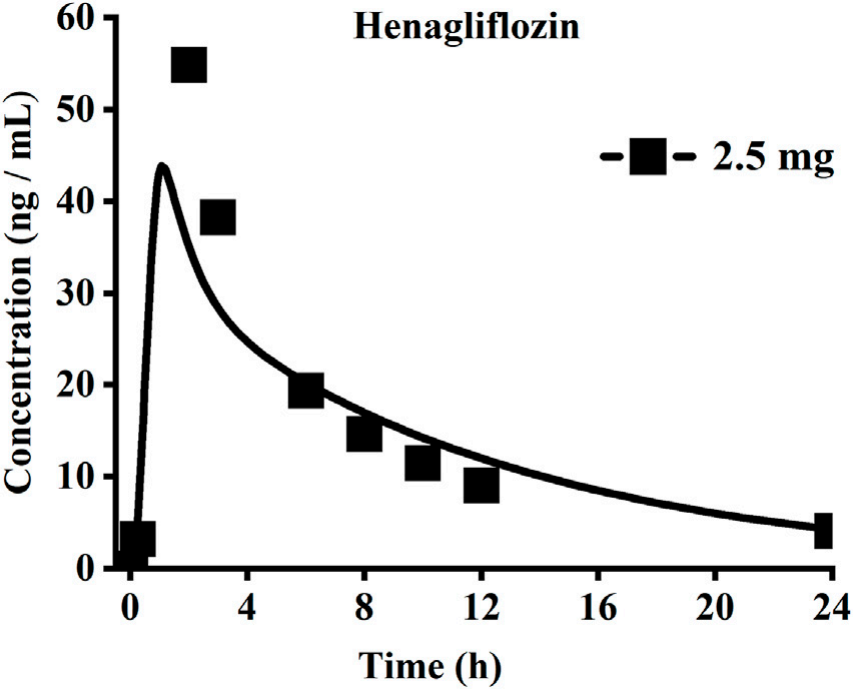
Simulated (lines) and observed (symbols) plasma concentrations for 2.5 mg single dose of henagliflozin with the original PBPK model.

**FIGURE 5:**
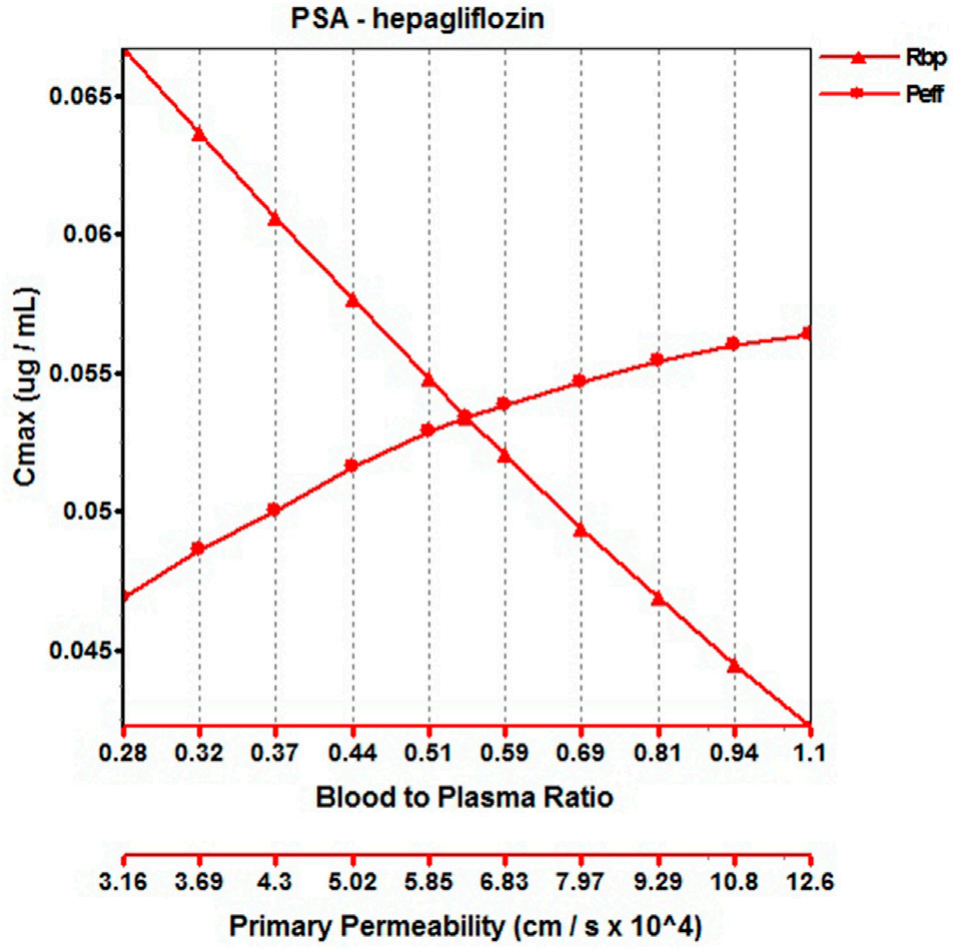
Parameter sensitivity analysis for 2.5 mg henagliflozin single oral administration. P_eff_, Permeability; Rbp, Blood to plasma ratio.

When the parameters were not optimized, the oral plasma profile of 2.5 mg appeared to have a lower C_max_ than the observed clinical data (Figure 4). To enhance the accuracy of the PBPK model, a sensitivity analysis was adopted to evaluate the key factors influencing the underprediction of C_max,_ such as solubility, effective permeability (peff), and the blood-to-plasma concentration ratio (Rbp), as well as the transit times of the stomach, small intestine, and colon. Due to the consistency of AUC between the observed and predicted values and the better fit of the clearance phase, plasma clearance was not considered in a sensitivity analysis. The results demonstrated that the C_max_ is highly sensitive to the changes in peff and Rbp (Figure 5). Because the model application depends on an accurate prediction of the exposure (C_max_ and AUC), we optimized Rbp and peff by fitting the observed values following a 2.5 mg dosage. According to the sensitivity analysis results, the Rbp ratio of 0.55 and Peff of 6.7 reasonably captured the oral plasma profile. Using the parameters from the best fit of henaglifloin 2.5 mg oral data, we simulated concentration-time curves of henagliflozin from 5 to 200 mg oral dose. The simulations rationally captured the observed pharmacokinetic data. However, the predicted C_max_ and T_max_ did not exactly match the observed values in high dosages. The optimized Rbp and peff led to correctly capturing C_max_. The optimized model captured the observed pharmacokinetic curves accurately (Figure 3). The final pharmacokinetic parameters for each oral dose and the assessment of fold error are summarized in Table 2, and the fold errors fell within 2-fold.

#### 3.1.4 Sotagliflozin

**FIGURE 6:**
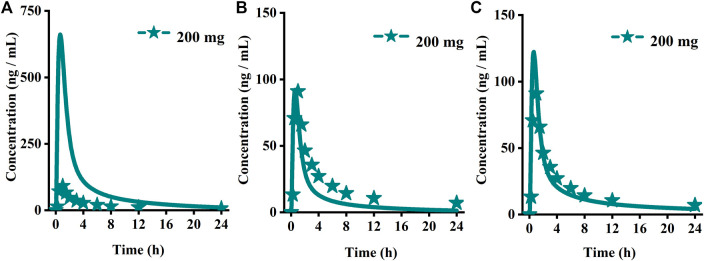
Simulated (lines) and observed (symbols) plasma concentrations for 200 mg single doses of sotagliflozin with the original PBPK model. **(A)** The origin PBPK model; **(B)** Optimized the gut first-pass effect; **(C)** Optimized the stomach transit time.

**FIGURE 7:**
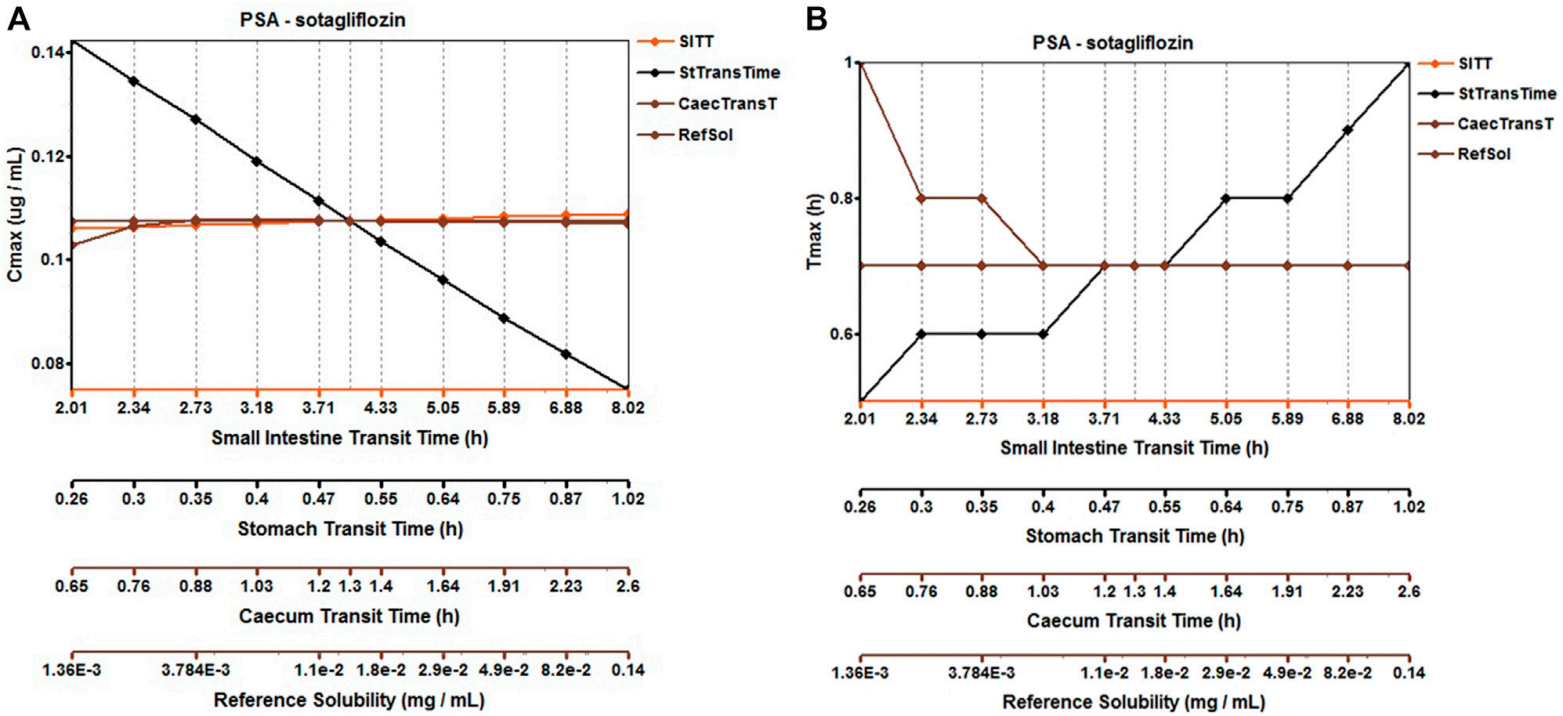
Parameter sensitivity analysis for 200 mg sotagliflozin single oral administration. PSA, Parameter sensitivity analysis; StTransTime, Stomach transit time; SITT, Small intestine transit time; Ref Sol, Reference solubility; CTranT, Colon transit time. **(A)** Parameter sensitivity analysis of the Cmax; **(B)** Parameter sensitivity analysis of the Tmax.

**FIGURE 8:**
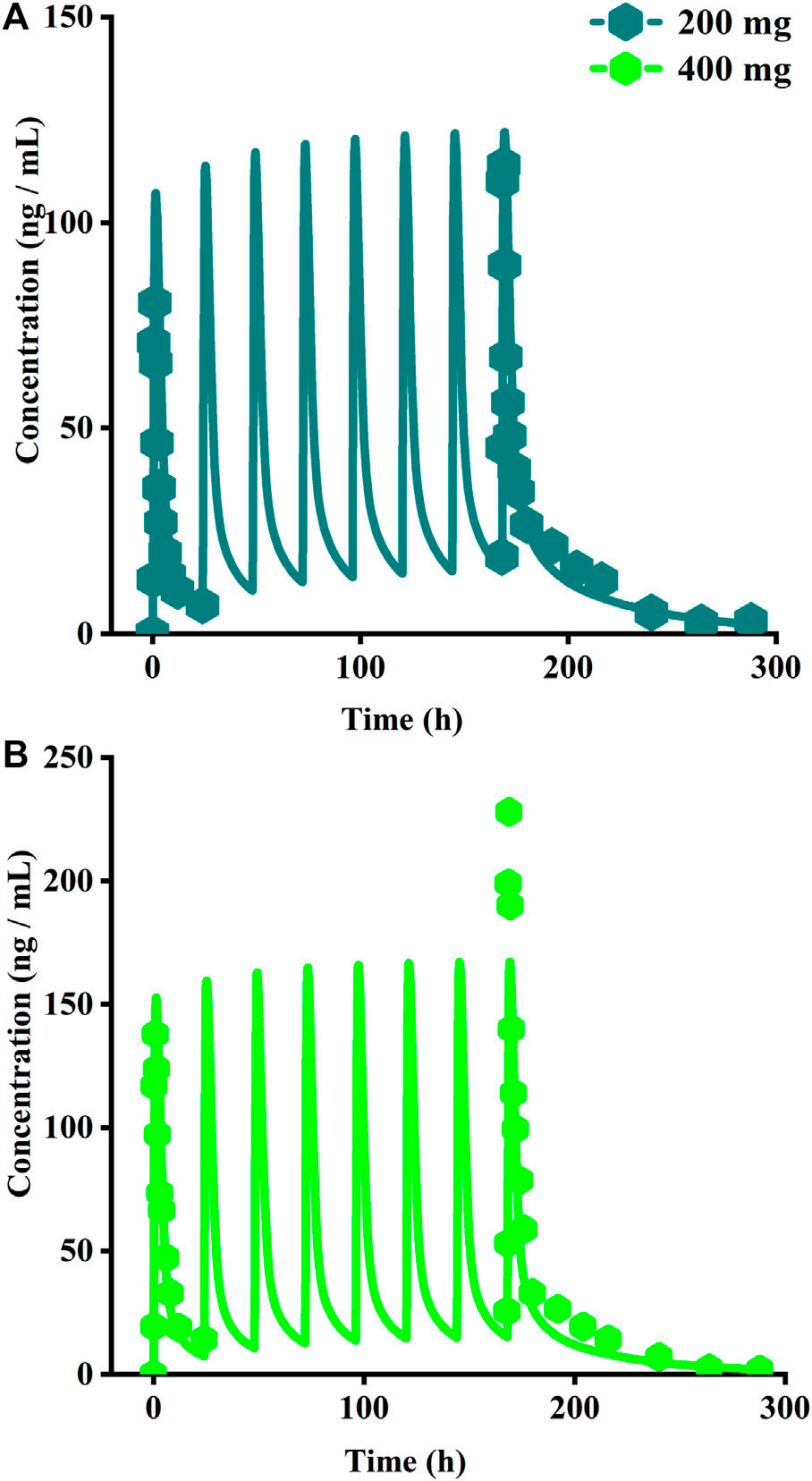
Simulated plasma concentrations of multiple doses (8 days doses) of sotagliflozin. **(A)** 200 mg once daily; **(B)** 400 mg once daily.

The original disposition parameters of sotagliflozin PBPK model were obtained from the ADMET predictor^TM^ module and internal data. However, as presented in Figure 6A, the initial simulated and observed plasma concentration-time curves are mismatched. Compared to the observed plasma concentration-time profile, the simulated oral lineshapes appeared to overestimate C_max_ and AUC and underestimate T_max_. Next, we refined and verified the model based on the steady-state mean plasma concentration-time curves. The developed PBPK model well simulated the observed clearance phase profile, and the total plasma clearance was close to the calculated clearance from the PK plus module. Therefore, essential factors were considered to optimize the oral absorption and distribution phase and improve model accuracy, such as solubility, intestinal transit time, P-gp efflux, chemical degradation, enterohepatic recirculation, and variable absorption across the gut (Peters, 2008). When co-administered with digoxin, the AUC_0-inf_ and C_max_ of digoxin increased by 27% and 52%, respectively, and we guessed that sotagliflozin inhibited P-gp (European Medicines Agency). Given the combined effect of P-gp, we optimized the gut first-pass effect to acquire the best fit for the observed concentration-time profile. The increase in the first pass effect lowered the simulated AUC, resulting in a slight underprediction of distribution. The V_ss_ calculated by the Lucaucas method appeared to be lower than that calculated by PK plus. Improving logP from 3.6 to 4.09 amplified the Kp values and corrected V_ss_ underprediction. The predicted results showed that the fold error of pharmacokinetic variables was within two folds. However, the lineshape was not well matched. To improve the accuracy of the model, we conducted a parameter sensitivity analysis to verify the influencing factor of the profile. Figure 7 shows that stomach transit time influences drug absorption significantly. Stomach transit time is an essential factor affecting absorption profile, and literature has noted that it varies greatly (Brophy et al., 1986; Petring and Flachs, 1990; Fruehauf et al., 2007). Therefore, optimizing the stomach transit time was necessary to estimate tissue concentrations better. The plasma concentration-time profile of sotagliflozin obtained from the groups of 400 mg was used to verify this optimized model. We further qualified the accuracy of the PBPK model based on the pharmacokinetic parameters of sotagliflozin from a clinical trial under multiple dosage regimens. The simulated oral plasma concentration-time profile corresponded with the observed one (Figures 5, 8), and the fold error between observed and predicted values fell within 2-fold. Therefore, a PBPK model for sotagliflozin was successfully developed and was able to describe *in vivo* target tissue concentrations reasonably.

**FIGURE 9:**
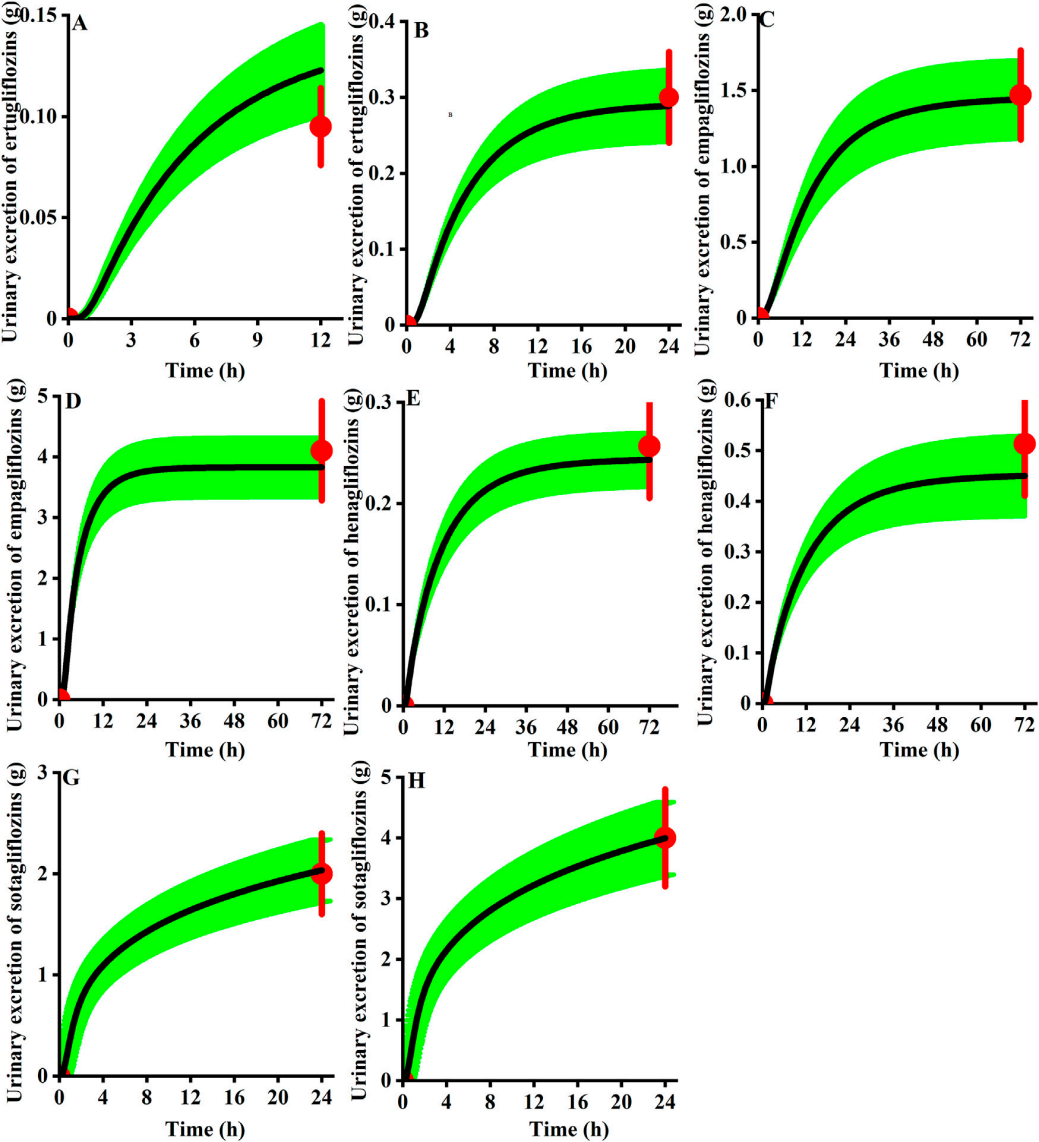
Observed urinary excretion (red) of gliflozins after administration and the 90th confidence interval of the virtual population simulation (green shadow, n = 100). **(A)** 5 mg ertugliflozin; **(B)** 15 mg ertugliflozin; **(C)** 10 mg empagliflozin; **(D)** 25 mg empagliflozin; **(E)** 5 mg henagliflozin; **(F)** 10 mg henagliflozin; **(G)** 200 mg sotagliflozin; **(H)** 400 mg sotagliflozin.

As presented in Figure 9, for the cumulative urine excretion profiles, we can find the clinical observation value within the 90% CI of the simulation in healthy virtual subjects at the clinical approval dose, demonstrating the accuracy of the mechanistic renal model.

### 3.2 Inhibition rate of SGLT 1 for intestinal glucose absorption

**FIGURE 10:**
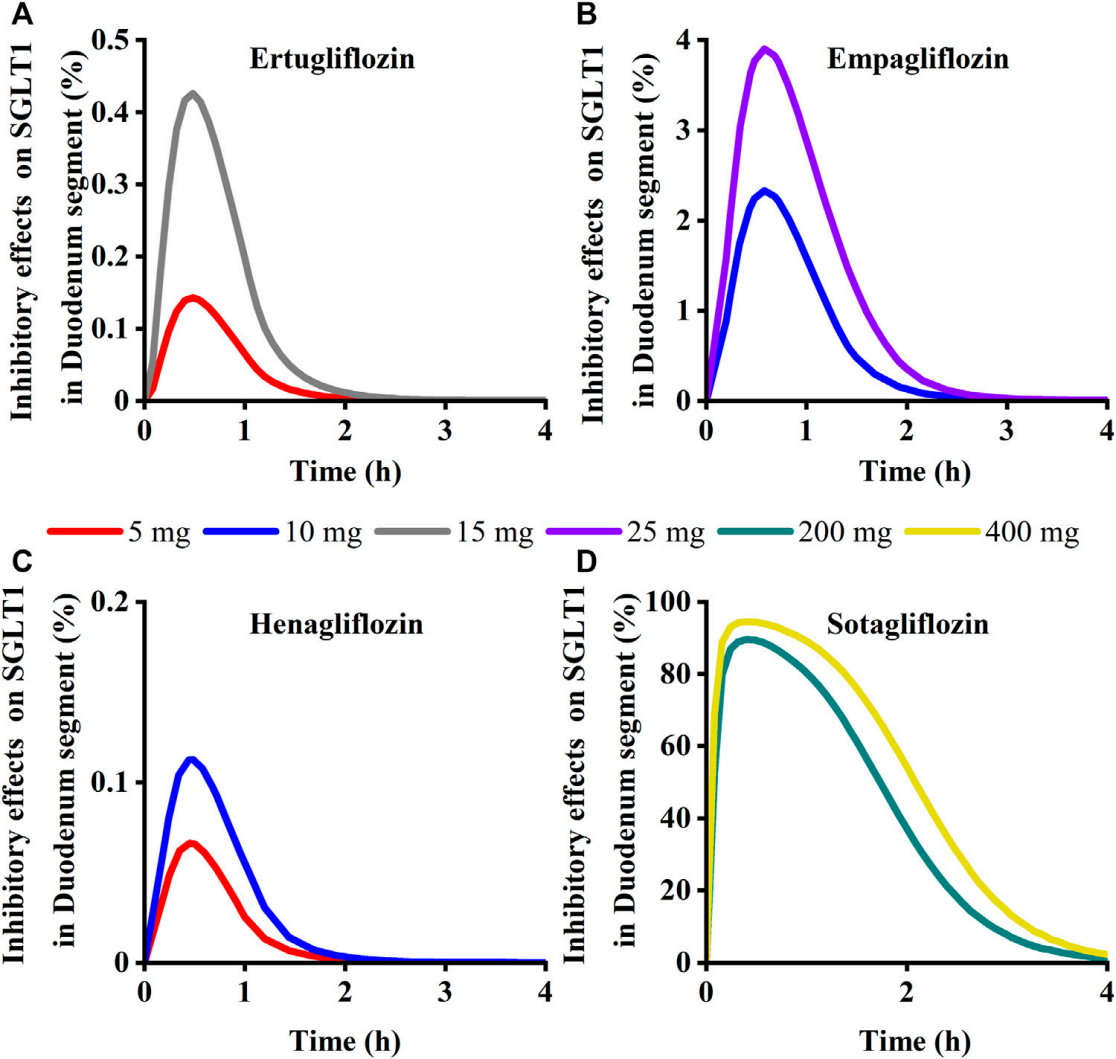
Simulation of inhibitory effects on sodium-glucose co-transporter1 (SGLT 1 s) in duodenum segment at approved doses. **(A)** Ertugliflozin; **(B)** Empagliflozin; **(C)** Henagliflozin; **(D)** Sotagliflozin.

**FIGURE 11:**
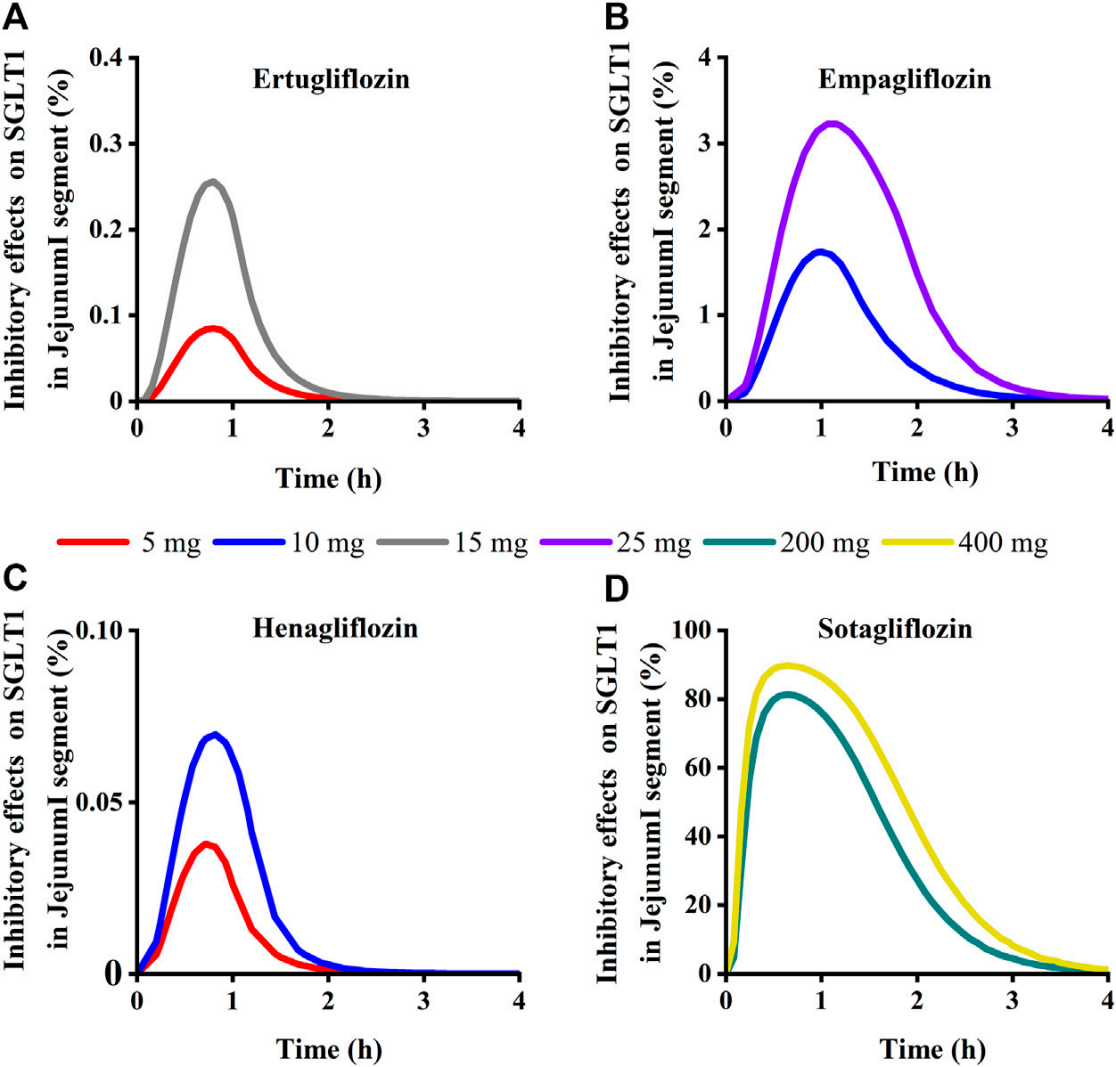
Simulation of inhibitory effects on sodium-glucose co-transporter1 (SGLT 1 s) in Jejunum I segment at approved doses. **(A)** Ertugliflozin; **(B)** Empagliflozin; **(C)** Henagliflozin; **(D)** Sotagliflozin.

To quantitatively assess the comparative inhibitory potencies of SGLT 1 in the absorption process, we simulated corresponding concentrations in plasma, duodenum, and upper jejunum (Jejunum I in the ACAT model) for each SGLT inhibitor at approved doses. Therefore, the SGLT 1 inhibition ratio was calculated based on the concentration. As shown in Figure 10; Figure 11, the suppression peaked in the 30 min following single-dose administration. The maximum inhibition rate of sotagliflozin in the duodenum and upper jejunum I remained higher (89%–94% and 89%–94%, respectively-Table 3). In contrast, 10 mg of empagliflozin had an inhibition ratio of just 1.7%–2.3%, whereas 10 mg henagliflozin and 15 mg ertugliflozin exhibited maximum inhibition rates of less than 0.5%, respectively. Compared to empagliflozin, sotaglifozin possessed a longer-lasting inhibitory effect on SGLT 1 transporter, and especially sotagliflozin can last for 5 h after oral administration (Figure 10; Figure 11).

**TABLE 3:** Maximum inhibition ratios on SGLT 1 in the intestine (duodenum and jejunum) and kidney.

Type	Ertugliflozin		Empagliflozin		Henagliflozin		Sotagliflozin	
	5 mg	10 mg	10 mg	25 mg	5 mg	10 mg	200 mg	400 mg
Duodenum	0.14	0.43	2.33	3.90	0.07	0.11	89.54	94.47
Jejunum I	0.09	0.26	1.74	3.22	0.04	0.07	81.41	89.75
Segment 3	0.09	0.26	0.89	1.91	0.04	0.08	6.66	13.05

### 3.3 Inhibition rate of SGLT 1 for renal glucose absorption

**FIGURE 12:**
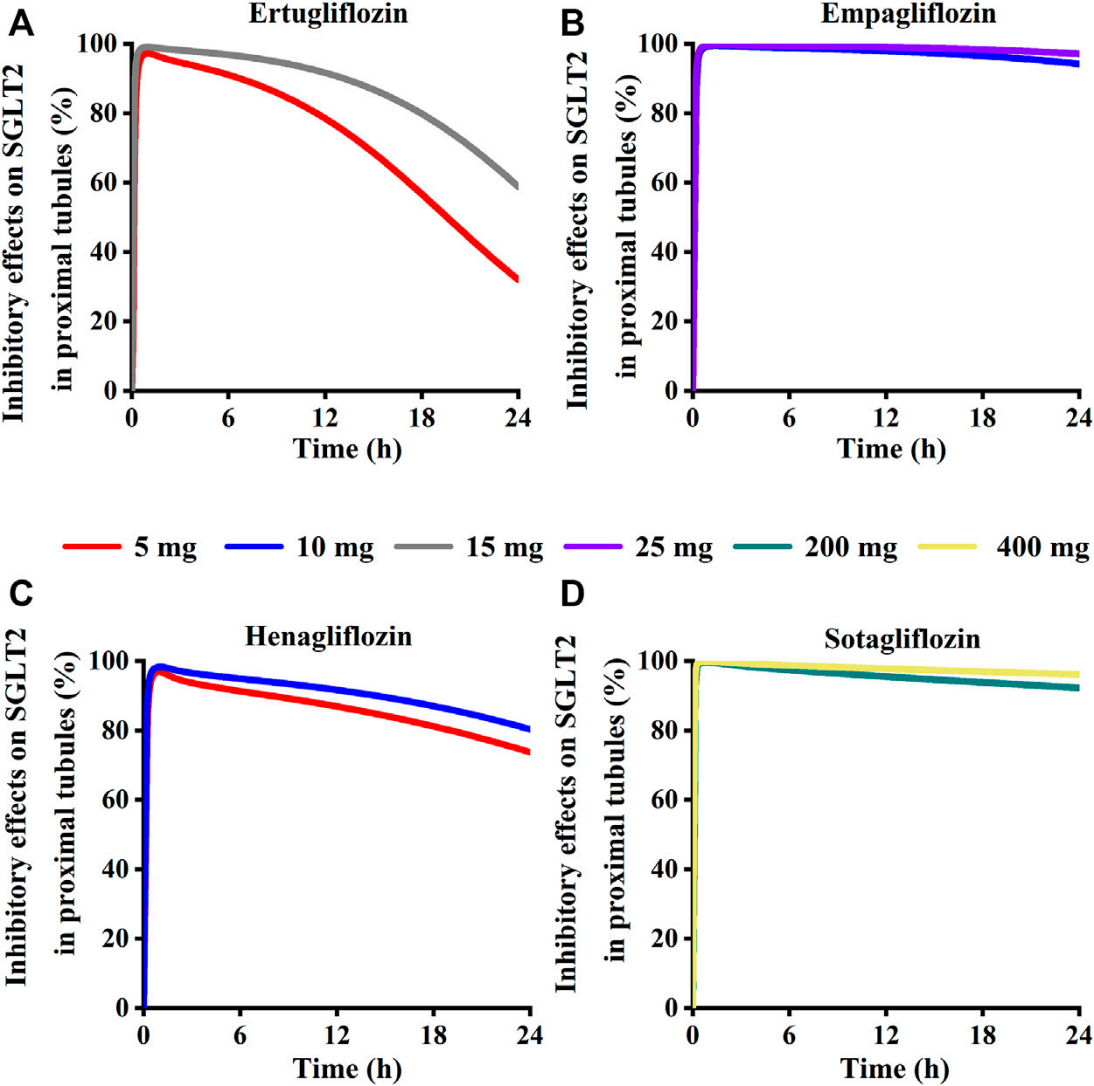
Simulation of inhibitory effects on sodium-glucose co-transporter2 (SGLT 2 s) in kidney proximal tubules at approved doses. **(A)** Ertugliflozin; **(B)** Empagliflozin; **(C)** Henagliflozin; **(D)** Sotagliflozin.

Simulation results showed that the maximum SGLT 2 inhibition ratio was approximately 100% at the highest approved dose of each gliflozin. As presented in Figure 12, model-predicted corresponding concentrations of each gliflozin after the highest administration doses could completely inhibit SGLT 2-mediate reabsorption. Thus, the reabsorption capacity of the SGLT 2 transporter was near saturated. The saturation of SGLT 2 leads to glucose influx into the S3 segment, and SGLT 1-driven glucose reabsorption plays a significant role in the kidney (Song et al., 2016). Therefore, it is particularly important to further evaluate the corresponding inhibitory effect of the SGLT 1 transporter.

**FIGURE 13:**
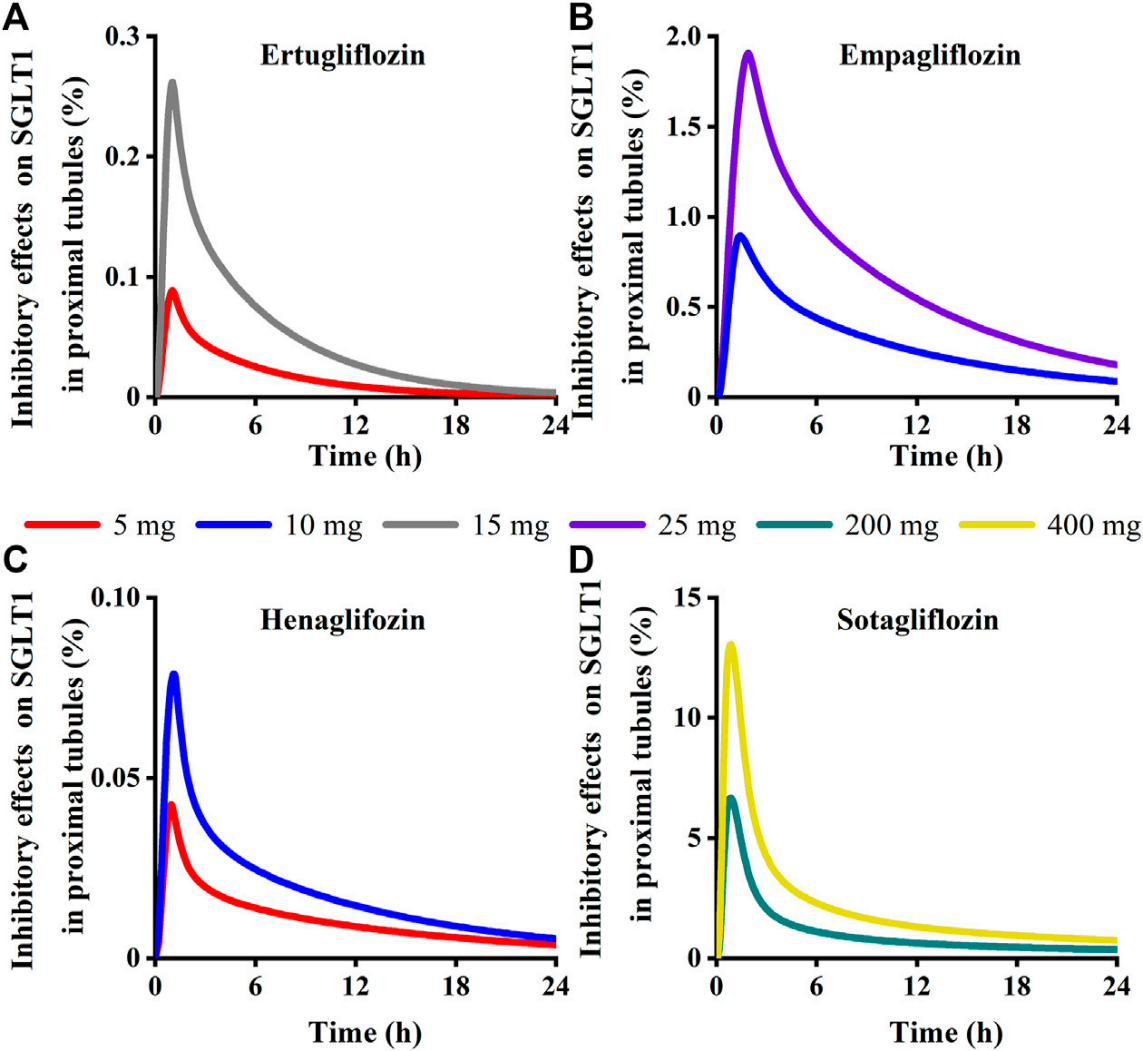
Simulation of inhibitory effects on sodium-glucose co-transporter1 (SGLT 1 s) in kidney proximal tubules at approved doses. **(A)** Ertugliflozin; **(B)** Empagliflozin; **(C)** Henagliflozin; **(D)** Sotagliflozin.

Model-predicted concentrations of each glifozin in the S3 segments were calculated, and the corresponding inhibition ratio on SGLT 1 was evaluated. Sotagliflozin has the maximum inhibitory effect at the approved dose (6.7%–13%) (Figure 13) (Table 3). In contrast, ertugliflozin, empagliflozin, and henagliflozin resulted in less than a 1% inhibition ratio in the S3 segment at the highest approved dose, especially henagliflozin. Simulations demonstrated that sotagliflozin was a potent SGLT1 inhibitor, whereas ertugliflozin, empagliflozin, and henagliflozin only exerted a weak inhibitory effect on SGLT1-mediated glucose reabsorption.

## 4 Discussion

The intestine and kidney are the targeted tissue of SGLT 2 inhibitors. SGLT 2 inhibitors have different inhibitory potencies on SGLT 1-mediated glucose absorption or reabsorption. Exploring the concentration in targeted sites was extremely critical for quantitatively analyzing the differences regarding inhibitory effects on SGLT1 transporter of ertugliflozin, empagliflozin, henagliflozin, and sotagliflozin.

Several mathematical models have been published to investigate SGLT-mediated renal glucose reabsorption, Mori *et al.* used a detailed PBPK model to demonstrate the renal SGLT1 inhibition capacity was less than 1% for both dapagliflozin and canagliflozin (Mori et al., 2016). Lu *et al.* developed a systems pharmacology model and simulated the SGLT1/2-mediated glucose reabsorption with and without SGLT2 inhibitor, simulation results proposed that when the SGLT 2 transporter is completely inhibited, the SGLT 1-dependent compensatory effect allows more glucose to be reabsorbed in the S3 segment (Lu et al., 2014). Balazki *et al.* first integrated the model of diabetes-associated renal hyperfiltration and PBPK model of dapagliflozin to confirm co-administration of renal SGLT1 inhibition is effective for reversing hyperfiltration compared to SGLT2 inhibition alone (Balazki et al., 2018). Yakovleva et al. (2019) used qantitative drug–disease systems model of dapagliflozin canagliflozin and empagliflozin to demonstrated that SGLT1-mediated transporter capacity was greater in T2DM. But we recognize that developed physiology models should not be expected to represent every detail. And taken together, these studies emphasize the importance of SGLT1 inhibition and understanding the comparative action of SGLT transporter for the treatment of T2DM. We analyzed the strengths of these SGLT pharmacology models to arrive at the PBPK structure used in our current study. We simulated the pharmacokinetic characteristics of empagliflozin, ertugliflozin, henagliflozin, and sotagliflozin. To further confirm the selectivity of SGLT1 and SGLT2 for these post-marketing SGLT2 inhibitors, we used Michaelis - Menten kinetics to described the action of glucose and SGLT transporter. The objective of our study was to quantitatively compare the relative action of SGLT1 and SGLT2 in each unstudied compound.

The current study demonstrated that using model-based prediction, we can successfully simulate the tissue distribution of each SGLT 2 inhibitor and describe its inhibitory action on the SGLT transporter in corresponding tissues, which is difficult to test experimentally. Based on the individual physiological, anatomical parameters, and physicochemical properties of drug, the developed PBPK model becomes significant to accurately characterize and forecast the tissue distribution of each gliflozin after administration (Sugimoto et al., 2020). The investigation offers the possibility of using the PBPK model to guide formulation development among similar drugs without conducting extensive clinical trials. Additionally, considering different SGLT transporter inhibitory activities would show different pharmacological outcomes, the developed model might act as a drug-disease platform for quantitative assessment of the inhibitory intensity of SGLT transporters in the intestine or kidney during treatment with SGLT 2 inhibitors in diverse dietary situations and T2DM patient sub-population, providing model-based dosage guidance.

The PBPK model for each gliflozin was initially developed and optimized using low-dose plasma concentration-time data. Then the optimized parameters and developed model were qualified by simulating other doses, and main factors affecting the oral pharmacokinetic curves were analyzed. When a permeability-limited model was used to describe the disposition of each gliflozin in the kidney, we found that the simulated and observed concentration-time profiles did not achieve a better fit. We chose the perfusion-limited model to illustrate the pharmacokinetic behaviors in the renal. In the population model, the simulated pharmacokinetic profiles matched the observed results, and the urinary excretion profile was well simulated by the filtration clearance (GFR × fup) in the renal model. Furthermore, the predicted concentration of each gliflozin of its parameters fell within two-fold errors. The results indicated the method could reasonably illustrate the ADME properties of each gliflozin in healthy volunteers. When GFR values were not published in clinical trials, we defined the mean GFR for 20–50 years as 127 mL**·**min-1/1.73 m^2^ adapted from Alcorn and McNamara (2002a) (Alcorn and McNamara, 2002). The original PBPK model requires being iteratively validated and optimized. The European Medicines Agency (EMA) encourages to analysis the uncertain parameters using PSA (Perry et al., 2020), which can present a range of uncertain model parameters relative to the pharmacokinetics. Based on the early fitting results, sensitivity analysis was conducted to assess the influence of model parameter variations on the simulated systemic exposure profiles. In the present study, the sensitive parameters are optimized by combining published literature and optimization module. A comprehensive evaluation of the optimized model supported the robustness of the plasma PBPK model.

In the small intestine, SGLT 1 and glucose transporter 2 (GLUT 2) are the significant transporters for glucose absorption (Koufakis et al., 2021). The potential advantage of SGLT2 inhibitors was their suppressing action on intestinal SGLT1, not only reducing the absorption of intestinal glucose but also promoting the secretion of peptide YY (PYY) and GLP-1 (Io et al., 2019) (Powell et al., 2020). The inhibition of intestinal SGLT1 transporter can exert a beneficial action on reducing postprandial hyperglycemia and improving high glucose concentration in T2DM patients (Powell et al., 2013). In the early stages of DM, lowering postprandial hyperglycemia is particularly important for managing chronic complications and overall glycemic control. Considering these points, dual SGLT 1/SGLT 2 inhibitors would enhance beneficial outcomes in DM and deserve further assessment. The disposition of SGLT2 inhibitor in the Duodenum and Jejunum I was predicted using the above developed PBPK model. Also, the inhibitory actions on intestinal SGLT1 were linked to the free concentration of gliflozins in the Duodenum and Jejunum I. Hence, the corresponding inhibition rate of SGLT 1 was further described at the clinically approved dose. Model-based prediction demonstrated that each SGLT 2 inhibitor exerted a postprandial glucose-lowing effect by inhibiting SGLT 1 to varying degrees in the intestine segment (Nishimura et al., 2015; Terra et al., 2017; Sims et al., 2018; Danne et al., 2019; Weng et al., 2021). The inhibition rate of sotagliflozin was 14% on intestinal SGLT1 3 h following single-dose administration of 400 mg, and the SGLT1 suppressant effect of sotagliflozin persisted for ≥3 h. These results suggest that, compared to the transient inhibitory effects of ertugliflozin, empagliflozin, and henagliflozin, the inhibitory effect of sotagliflozin was long-lasting on intestinal SGLT1. The predicted sotagliflozin concentrations in the Jejunum Ⅰ segment 3 h following oral single-dose administration of 400 mg was 0.0173 mg/mL, more than 230-fold higher than that in plasma (73 ng/mL). According to these results, the sustained inhibitory effect of sotagliflozin on SGLT1 depends on its high concentration in the intestinal segment instead of that in plasma. This conclusion is consistent with studies (Zambrowicz et al., 2012; Powell et al., 2020).

Inhibition of the SGLT1 transporter is associated with osmotic diarrhea, malabsorption, and metabolic acidosis (Tsimihodimos et al., 2018). However, no signs of adverse metabolic acidosis and GI effects were reported in T2DM patients treated with a single dose of 400 mg of sotagliflozin (Lehmann and Hornby, 2016). The recent meta-analysis about the safety and tolerability of sotagliflozin recommended the 400 mg dose as a clinical dose, indicating that the adverse events of sotagliflozin were acceptable to diabetes patients (Zhou et al., 2022). Therefore, as long as there are no significantly associated side effects, the dual SGLT 1/SGLT 2 inhibition remains an option for formulation development and clinical medication therapy. Meanwhile, more effective dual SGLT 1/SGLT 2 inhibitors can be discovered by combining clinical trials and PBPK model prediction approaches.

Although urine excretion of these four gliflozins is low, the renal proximal tubular concentration of free drugs is sufficient to express pharmacological activities. All four gliflozins have a strong renal SGLT2 inhibitory effect at their clinical dose. In contrast, neither henagliflozin, ertugliflozin, nor empagliflozin actively inhibited SGLT1-mediated glucose reabsorption. Only sotagliflozin exhibited sustained exposures, with 6.7%–13% inhibition on SGLT 1 in the S3 segment, increasing glucose excretion in T2DM patients. The results of clinical trials of sotagliflozin single-dose and multiple-dose in the Chinese population suggest that sotaglifliozin can lower plasma glucose and is generally well tolerated (He et al., 2022). Gliflozin molecules occupy the SGLT2 transporter at clinical dosage (inhibition rate to 99% at peak exposure), and the contribution of SGLT2 is suppressed, resulting in the buildup of glucose in the S3 segment of the proximal tubules. The activity of SGLT1-mediated glucose reabsorption is intense (Lu et al., 2014). The results of modeling and simulation confirmed this concept. Moreover, more stronger inhibition of SGLT 2 could lead to a greater risk of genitourinary tract infections, pre-renal failure, and euglycemic diabetic ketoacidosis (Tsimihodimos et al., 2018). However, concomitant SGLT1 inhibition may reduce the risk of acute renal damage induced by SGLT 2 inhibitors (Dominguez Rieg and Rieg, 2019). Sotagliflozin inhibits SGLT1 more strongly than those, so it may significantly promote urinary glucose excretion (UGE). Sotagliflozin remained distinct due to its pharmacokinetic characteristics, plasma protein binding, and higher selectivity for SGLT1 than the other three gliflozins. Although sotagliflozin has a long acting and strong inhibitory effect on renal SGLT1, the mean UGE was not satisfactory in the clinical trial, and we guess that the sustained inhibition of sotagliflozin on intestinal SGLT1 caused the strong effect on PPG, resulting in the lower UGE produced by sotagliflozin (Takebayashi and Inukai, 2017).

In this study, we developed and qualified the PBPK model for ertugliflozin, empagliflozin, henagliflozin, and sotagliflozin. Meanwhile, we used the PBPK model to predict drug concentration-time profiles in the target tissue quantitatively. Following a range of doses, the model accurately described the single-dose and multiple-dose blood exposure profiles of each gliflozin. According to our predictions, therapeutic dosages of ertugliflozin, empagliflozin, henagliflozin, and sotagliflozin almost completely inhibit SGLT 2. Ertugliflozin, empagliflozin, and henagliflozin have a weak inhibitory effect on SGLT 1, and sotagliflozin has a more potent and longer-lasting inhibitory effect on SGLT 1. This study aimed to quantify the inhibitory effects of gliflozins on intestinal and renal. However, SGLT transporters also are expressed in the central nervous system, and the SGLT 1 transporters are related to protecting against ischemia/reperfusion brain injury (Pawlos et al., 2021). Also, gliflozins are fat-soluble and cross the blood-brain barrier, inhibiting acetylcholinesterase (AChE) and improving cognitive function (Hierro-Bujalance et al., 2020). In conclusion, SGLT inhibitors improve impaired cognitive function in diabetic patients with atherosclerosis by inhibiting SGLT transporters in the brain. In addition, SGLT-2 inhibitors have benefits on cardiovascular in patients with T2DM, as proven by several key outcomes trials, including VERTIS CV (ertugliflozin) (Rao, 2022), EMPA-REG OUTCOME (empagliflozin), and SCORED (sotagliflozin) (Halimi, 2021). FDA authorized empagliflozin for the treatment of heart failure. Although the specific mechanism of action is not fully established, some evidence suggests that SGLT 1 activity significantly increases in diabetic heart compared to healthy subjects (Maejima, 2019). Therefore, quantifying the inhibitory effect on SGLT transporters in the brain and heart is meaningful for further understanding the effect of neuroprotection (Pawlos et al., 2021) and cardioprotection.

## Data Availability

The original contributions presented in the study are included in the article/supplementary material, further inquiries can be directed to the corresponding author.

## References

[B1] Alcorn J. McNamara P. J. (2002). Ontogeny of hepatic and renal systemic clearance pathways in infants: Part I. Clin. Pharmacokinet. 41 (12), 959–998. 10.2165/00003088-200241120-00003 12222995

[B2] Bailey C. J. (2011). Renal glucose reabsorption inhibitors to treat diabetes. Trends Pharmacol. Sci. 32 (2), 63–71. 10.1016/j.tips.2010.11.011 21211857

[B3] Balazki P. Schaller S. Eissing T. Lehr T. (2018). A quantitative systems pharmacology kidney model of diabetes associated renal hyperfiltration and the effects of SGLT inhibitors. CPT Pharmacometrics Syst. Pharmacol. 7 (12), 788–797. 10.1002/psp4.12359 30270578 PMC6310870

[B4] Brophy C. M. Moore J. G. Christian P. E. Egger M. J. Taylor A. T. (1986). Variability of gastric emptying measurements in man employing standardized radiolabeled meals. Dig. Dis. Sci. 31 (8), 799–806. 10.1007/bf01296046 3731973

[B5] Buse J. B. Wexler D. J. Tsapas A. Rossing P. Mingrone G. Mathieu C. (2020). 2019 update to: Management of hyperglycemia in type 2 diabetes, 2018. A consensus report by the American diabetes association (ADA) and the European association for the study of diabetes (easd). Diabetes Care 43 (2), 487–493. 10.2337/dci19-0066 31857443 PMC6971782

[B6] Callegari E. Lin J. Tse S. Goosen T. C. Sahasrabudhe V. (2021). Physiologically-based pharmacokinetic modeling of the drug-drug interaction of the UGT substrate ertugliflozin following Co-administration with the UGT inhibitor mefenamic acid. CPT Pharmacometrics Syst. Pharmacol. 10 (2), 127–136. 10.1002/psp4.12581 33314761 PMC7894401

[B7] Cefalo C. M. A. Cinti F. Moffa S. Impronta F. Sorice G. P. Mezza T. (2019). Sotagliflozin, the first dual SGLT inhibitor: Current outlook and perspectives. Cardiovasc Diabetol. 18 (1), 20. 10.1186/s12933-019-0828-y 30819210 PMC6393994

[B8] Chen L. Z. Jungnik A. Mao Y. Philip E. Sharp D. Unseld A. (2015). Biotransformation and mass balance of the SGLT2 inhibitor empagliflozin in healthy volunteers. Xenobiotica 45 (6), 520–529. 10.3109/00498254.2014.999141 25547626

[B9] Danne T. Cariou B. Buse J. B. Garg S. K. Rosenstock J. Banks P. (2019). Improved time in range and glycemic variability with sotagliflozin in combination with insulin in adults with type 1 diabetes: A pooled analysis of 24-week continuous glucose monitoring data from the inTandem program. Diabetes Care 42 (5), 919–930. 10.2337/dc18-2149 30833371 PMC6905498

[B10] Davies M. J. Aroda V. R. Collins B. S. Gabbay R. A. Green J. Maruthur N. M. (2022). Management of hyperglycaemia in type 2 diabetes, 2022. A consensus report by the American diabetes association (ADA) and the European association for the study of diabetes (easd). Diabetologia 65 (12), 1925–1966. 10.1007/s00125-022-05787-2 36151309 PMC9510507

[B11] Dawra V. K. Liang Y. Shi H. Bass A. Hickman A. Terra S. G. (2019). A PK/PD study comparing twice-daily to once-daily dosing regimens of ertugliflozin in healthy subjects. Int. J. Clin. Pharmacol. Ther. 57 (4), 207–216. 10.5414/cp203343 30802200 PMC6528385

[B12] De Buck S. S. Sinha V. K. Fenu L. A. Gilissen R. A. Mackie C. E. Nijsen M. J. (2007). The prediction of drug metabolism, tissue distribution, and bioavailability of 50 structurally diverse compounds in rat using mechanism-based absorption, distribution, and metabolism prediction tools. Drug Metab. Dispos. 35 (4), 649–659. 10.1124/dmd.106.014027 17267621

[B13] DeFronzo R. A. Davidson J. A. Del Prato S. (2012). The role of the kidneys in glucose homeostasis: A new path towards normalizing glycaemia. Diabetes Obes. Metab. 14 (1), 5–14. 10.1111/j.1463-1326.2011.01511.x 21955459

[B14] Demin O. Jr. Yakovleva T. Kolobkov D. Demin O. (2014). Analysis of the efficacy of SGLT2 inhibitors using semi-mechanistic model. Front. Pharmacol. 5, 218. 10.3389/fphar.2014.00218 25352807 PMC4195280

[B15] Dominguez Rieg J. A. Rieg T. (2019). What does sodium-glucose co-transporter 1 inhibition add: Prospects for dual inhibition. Diabetes Obes. Metab. 21, 43–52. 10.1111/dom.13630 PMC6516085 31081587

[B16] Emami Riedmaier A. Burt H. Abduljalil K. Neuhoff S. (2016). More power to OATP1B1: An evaluation of sample size in pharmacogenetic studies using a rosuvastatin PBPK model for intestinal, hepatic, and renal transporter-mediated clearances. J. Clin. Pharmacol. 56, S132–S142. 10.1002/jcph.669 27385171 PMC5096019

[B17] Fediuk D. J. Nucci G. Dawra V. K. Cutler D. L. Amin N. B. Terra S. G. (2020). Overview of the clinical pharmacology of ertugliflozin, a novel sodium-glucose cotransporter 2 (SGLT2) inhibitor. Clin. Pharmacokinet. 59 (8), 949–965. 10.1007/s40262-020-00875-1 32337660 PMC7403171

[B18] Fruehauf H. Goetze O. Steingoetter A. Kwiatek M. Boesiger P. Thumshirn M. (2007). Intersubject and intrasubject variability of gastric volumes in response to isocaloric liquid meals in functional dyspepsia and health. Neurogastroenterol. Motil. 19 (7), 553–561. 10.1111/j.1365-2982.2007.00904.x 17593136

[B19] Garcia-Ropero A. Badimon J. J. Santos-Gallego C. G. (2018). The pharmacokinetics and pharmacodynamics of SGLT2 inhibitors for type 2 diabetes mellitus: The latest developments. Expert Opin. Drug Metab. Toxicol. 14 (12), 1287–1302. 10.1080/17425255.2018.1551877 30463454

[B20] Grempler R. Thomas L. Eckhardt M. Himmelsbach F. Sauer A. Sharp D. E. (2012). Empagliflozin, a novel selective sodium glucose cotransporter-2 (SGLT-2) inhibitor: Characterisation and comparison with other SGLT-2 inhibitors. Diabetes Obes. Metab. 14 (1), 83–90. 10.1111/j.1463-1326.2011.01517.x 21985634

[B21] Halimi J. M. (2021). SGLT2 inhibitors: A new era for our patients. Nephrol. Ther. 17 (3), 143–148. 10.1016/j.nephro.2020.12.006 33773943

[B22] He X. Gao X. Xie P. Liu Y. Bai W. Liu Y. (2022). Pharmacokinetics, pharmacodynamics, safety and tolerability of sotagliflozin after multiple ascending doses in Chinese healthy subjects. Drug Des. Devel Ther. 16, 2967–2980. 10.2147/dddt.S372575 PMC9464004 36097559

[B23] Hierro-Bujalance C. Infante-Garcia C. Del Marco A. Herrera M. Carranza-Naval M. J. Suarez J. (2020). Empagliflozin reduces vascular damage and cognitive impairment in a mixed murine model of Alzheimer's disease and type 2 diabetes. Alzheimers Res. Ther. 12 (1), 40. 10.1186/s13195-020-00607-4 32264944 PMC7140573

[B24] Io F. Gunji E. Koretsune H. Kato K. Sugisaki-Kitano M. Okumura-Kitajima L. (2019). SGL5213, a novel and potent intestinal SGLT1 inhibitor, suppresses intestinal glucose absorption and enhances plasma GLP-1 and GLP-2 secretion in rats. Eur. J. Pharmacol. 853, 136–144. 10.1016/j.ejphar.2019.03.023 30878385

[B25] Jones H. M. Parrott N. Jorga K. Lavé T. (2006). A novel strategy for physiologically based predictions of human pharmacokinetics. Clin. Pharmacokinet. 45 (5), 511–542. 10.2165/00003088-200645050-00006 16640456

[B26] Kong W. M. Sun B. B. Wang Z. J. Zheng X. K. Zhao K. J. Chen Y. (2020). Physiologically based pharmacokinetic-pharmacodynamic modeling for prediction of vonoprazan pharmacokinetics and its inhibition on gastric acid secretion following intravenous/oral administration to rats, dogs and humans. Acta Pharmacol. Sin. 41 (6), 852–865. 10.1038/s41401-019-0353-2 31969689 PMC7468366

[B27] Koufakis T. Metallidis S. Zebekakis P. Kotsa K. (2021). Intestinal SGLT1 as a therapeutic target in COVID-19-related diabetes: A "two-edged sword" hypothesis. Br. J. Clin. Pharmacol. 87 (10), 3643–3646. 10.1111/bcp.14800 33684969 PMC8251113

[B28] Lehmann A. Hornby P. J. (2016). Intestinal SGLT1 in metabolic health and disease. Am. J. Physiol. Gastrointest. Liver Physiol. 310 (11), G887–G898. 10.1152/ajpgi.00068.2016 27012770

[B29] Li Y. Mu Y. Shi H. Liang Y. Liu Z. Matschke K. (2020). Pharmacokinetic properties of single and multiple doses of ertugliflozin, a selective inhibitor of SGLT2, in healthy Chinese subjects. Clin. Pharmacol. Drug Dev. 9 (1), 97–106. 10.1002/cpdd.686 30934166 PMC7003779

[B30] Li Y. Nucci G. Yamamoto Y. Fediuk D. J. Sahasrabudhe V. (2021). Pharmacokinetics and pharmacodynamics of ertugliflozin in healthy Japanese and western subjects. Clin. Pharmacol. Drug Dev. 10 (7), 765–776. 10.1002/cpdd.908 33434408 PMC8359436

[B31] Lu Y. Griffen S. C. Boulton D. W. Leil T. A. (2014). Use of systems pharmacology modeling to elucidate the operating characteristics of SGLT1 and SGLT2 in renal glucose reabsorption in humans. Front. Pharmacol. 5, 274. 10.3389/fphar.2014.00274 25540623 PMC4261707

[B32] Maejima Y. (2019). SGLT2 inhibitors play a salutary role in heart failure via modulation of the mitochondrial function. Front. Cardiovasc Med. 6, 186. 10.3389/fcvm.2019.00186 31970162 PMC6960132

[B33] Mascitti V. Maurer T. S. Robinson R. P. Bian J. Boustany-Kari C. M. Brandt T. (2011). Discovery of a clinical candidate from the structurally unique dioxa-bicyclo[3.2.1]octane class of sodium-dependent glucose cotransporter 2 inhibitors. J. Med. Chem. 54 (8), 2952–2960. 10.1021/jm200049r 21449606

[B34] Meng W. Ellsworth B. A. Nirschl A. A. McCann P. J. Patel M. Girotra R. N. (2008). Discovery of dapagliflozin: A potent, selective renal sodium-dependent glucose cotransporter 2 (SGLT2) inhibitor for the treatment of type 2 diabetes. J. Med. Chem. 51 (5), 1145–1149. 10.1021/jm701272q 18260618

[B35] Mori K. Saito R. Nakamaru Y. Shimizu M. Yamazaki H. (2016). Physiologically based pharmacokinetic-pharmacodynamic modeling to predict concentrations and actions of sodium-dependent glucose transporter 2 inhibitor canagliflozin in human intestines and renal tubules. Biopharm. Drug Dispos. 37 (8), 491–506. 10.1002/bdd.2040 27604638

[B36] Moss D. M. Siccardi M. (2014). Optimizing nanomedicine pharmacokinetics using physiologically based pharmacokinetics modelling. Br. J. Pharmacol. 171 (17), 3963–3979. 10.1111/bph.12604 24467481 PMC4243971

[B37] Nishimura R. Tanaka Y. Koiwai K. Inoue K. Hach T. Salsali A. (2015). Effect of empagliflozin monotherapy on postprandial glucose and 24-hour glucose variability in Japanese patients with type 2 diabetes mellitus: A randomized, double-blind, placebo-controlled, 4-week study. Cardiovasc Diabetol. 14, 11. 10.1186/s12933-014-0169-9 25633683 PMC4339254

[B38] Parrott N. Paquereau N. Coassolo P. Lavé T. (2005). An evaluation of the utility of physiologically based models of pharmacokinetics in early drug discovery. J. Pharm. Sci. 94 (10), 2327–2343. 10.1002/jps.20419 16136543

[B39] Pawlos A. Broncel M. Woźniak E. Gorzelak-Pabiś P. (2021). Neuroprotective effect of SGLT2 inhibitors. Molecules 26 (23). 10.3390/molecules26237213 PMC8659196 34885795

[B40] Perry C. Davis G. Conner T. M. Zhang T. (2020). Utilization of physiologically based pharmacokinetic modeling in clinical pharmacology and therapeutics: An overview. Curr. Pharmacol. Rep. 6 (3), 71–84. 10.1007/s40495-020-00212-x 32399388 PMC7214223

[B41] Peters S. A. (2008). Identification of intestinal loss of a drug through physiologically based pharmacokinetic simulation of plasma concentration-time profiles. Clin. Pharmacokinet. 47 (4), 245–259. 10.2165/00003088-200847040-00003 18336054

[B42] Petring O. U. Flachs H. (1990). Inter- and intrasubject variability of gastric emptying in healthy volunteers measured by scintigraphy and paracetamol absorption. Br. J. Clin. Pharmacol. 29 (6), 703–708. 10.1111/j.1365-2125.1990.tb03691.x 2378789 PMC1380172

[B43] Powell D. R. Smith M. Greer J. Harris A. Zhao S. DaCosta C. (2013). LX4211 increases serum glucagon-like peptide 1 and peptide YY levels by reducing sodium/glucose cotransporter 1 (SGLT1)-mediated absorption of intestinal glucose. J. Pharmacol. Exp. Ther. 345 (2), 250–259. 10.1124/jpet.113.203364 23487174

[B44] Powell D. R. Zambrowicz B. Morrow L. Beysen C. Hompesch M. Turner S. (2020). Sotagliflozin decreases postprandial glucose and insulin concentrations by delaying intestinal glucose absorption. J. Clin. Endocrinol. Metab. 105 (4), e1235–e1249. 10.1210/clinem/dgz258 31837264 PMC7067537

[B45] Rao S. (2022). Use of sodium-glucose cotransporter-2 inhibitors in clinical practice for heart failure prevention and treatment: Beyond type 2 diabetes. A narrative review. Adv. Ther. 39 (2), 845–861. 10.1007/s12325-021-01989-z 34881413 PMC8866261

[B46] Rodgers T. Leahy D. Rowland M. (2005). Physiologically based pharmacokinetic modeling 1: Predicting the tissue distribution of moderate-to-strong bases. J. Pharm. Sci. 94 (6), 1259–1276. 10.1002/jps.20322 15858854

[B47] Rodgers T. Rowland M. (2006). Physiologically based pharmacokinetic modelling 2: Predicting the tissue distribution of acids, very weak bases, neutrals and zwitterions. J. Pharm. Sci. 95 (6), 1238–1257. 10.1002/jps.20502 16639716

[B48] Sager J. E. Yu J. Ragueneau-Majlessi I. Isoherranen N. (2015). Physiologically based pharmacokinetic (PBPK) modeling and simulation approaches: A systematic review of published models, applications, and model verification. Drug Metab. Dispos. 43 (11), 1823–1837. 10.1124/dmd.115.065920 26296709 PMC4613950

[B49] Samukawa Y. Mutoh M. Chen S. Mizui N. (2017). Mechanism-based pharmacokinetic-pharmacodynamic modeling of luseogliflozin, a sodium glucose Co-transporter 2 inhibitor, in Japanese patients with type 2 diabetes mellitus. Biol. Pharm. Bull. 40 (8), 1207–1218. 10.1248/bpb.b16-00998 28769002

[B50] Sarashina A. Koiwai K. Seman L. J. Yamamura N. Taniguchi A. Negishi T. (2013). Safety, tolerability, pharmacokinetics and pharmacodynamics of single doses of empagliflozin, a sodium glucose cotransporter 2 (SGLT2) inhibitor, in healthy Japanese subjects. Drug Metab. Pharmacokinet. 28 (3), 213–219. 10.2133/dmpk.dmpk-12-rg-082 23149871

[B51] Scheen A. J. (2015). Pharmacodynamics, efficacy and safety of sodium-glucose co-transporter type 2 (SGLT2) inhibitors for the treatment of type 2 diabetes mellitus. Drugs 75 (1), 33–59. 10.1007/s40265-014-0337-y 25488697

[B52] Sims H. Smith K. H. Bramlage P. Minguet J. (2018). Sotagliflozin: A dual sodium-glucose co-transporter-1 and -2 inhibitor for the management of type 1 and type 2 diabetes mellitus. Diabet. Med. 35 (8), 1037–1048. 10.1111/dme.13645 29637608

[B53] Sokolov V. Yakovleva T. Chu L. Tang W. Greasley P. J. Johansson S. (2020). Differentiating the sodium-glucose cotransporter 1 inhibition capacity of canagliflozin vs. Dapagliflozin and empagliflozin using quantitative systems pharmacology modeling. CPT Pharmacometrics Syst. Pharmacol. 9 (4), 222–229. 10.1002/psp4.12498 32064793 PMC7180004

[B54] Song P. Onishi A. Koepsell H. Vallon V. (2016). Sodium glucose cotransporter SGLT1 as a therapeutic target in diabetes mellitus. Expert Opin. Ther. Targets 20 (9), 1109–1125. 10.1517/14728222.2016.1168808 26998950 PMC5045806

[B55] Sugimoto H. Chen S. Qian M. G. (2020). Pharmacokinetic characterization and tissue distribution of fusion protein therapeutics by orthogonal bioanalytical assays and minimal PBPK modeling. Molecules 25 (3). 10.3390/molecules25030535 PMC7037219 31991858

[B56] Takebayashi K. Inukai T. (2017). Effect of sodium glucose cotransporter 2 inhibitors with low SGLT2/SGLT1 selectivity on circulating glucagon-like peptide 1 levels in type 2 diabetes mellitus. J. Clin. Med. Res. 9 (9), 745–753. 10.14740/jocmr3112w 28811850 PMC5544478

[B57] Terra S. G. Focht K. Davies M. Frias J. Derosa G. Darekar A. (2017). Phase III, efficacy and safety study of ertugliflozin monotherapy in people with type 2 diabetes mellitus inadequately controlled with diet and exercise alone. Diabetes Obes. Metab. 19 (5), 721–728. 10.1111/dom.12888 28116776

[B58] Tonneijck L. Muskiet M. H. Smits M. M. van Bommel E. J. Heerspink H. J. van Raalte D. H. (2017). Glomerular hyperfiltration in diabetes: Mechanisms, clinical significance, and treatment. J. Am. Soc. Nephrol. 28 (4), 1023–1039. 10.1681/asn.2016060666 28143897 PMC5373460

[B59] Tsimihodimos V. Filippas-Ntekouan S. Elisaf M. (2018). SGLT1 inhibition: Pros and cons. Eur. J. Pharmacol. 838, 153–156. 10.1016/j.ejphar.2018.09.019 30240793

[B60] Wang L. Wu C. Shen L. Liu H. Chen Y. Liu F. (2016). Evaluation of drug-drug interaction between henagliflozin, a novel sodium-glucose co-transporter 2 inhibitor, and metformin in healthy Chinese males. Xenobiotica 46 (8), 703–708. 10.3109/00498254.2015.1113576 26608671

[B61] Wang Y. Lou Y. Wang J. Li D. Chen H. Zheng T. (2019). Design, synthesis and biological evaluation of 6-deoxy O-spiroketal C-arylglucosides as novel renal sodium-dependent glucose cotransporter 2 (SGLT2) inhibitors for the treatment of type 2 diabetes. Eur. J. Med. Chem. 180, 398–416. 10.1016/j.ejmech.2019.07.032 31325786

[B62] Weng J. Zeng L. Zhang Y. Qu S. Wang X. Li P. (2021). Henagliflozin as add-on therapy to metformin in patients with type 2 diabetes inadequately controlled with metformin: A multicentre, randomized, double-blind, placebo-controlled, phase 3 trial. Diabetes Obes. Metab. 23 (8), 1754–1764. 10.1111/dom.14389 33769656

[B63] Yakovleva T. Sokolov V. Chu L. Tang W. Greasley P. J. Peilot Sjögren H. (2019). Comparison of the urinary glucose excretion contributions of SGLT2 and SGLT1: A quantitative systems pharmacology analysis in healthy individuals and patients with type 2 diabetes treated with SGLT2 inhibitors. Diabetes Obes. Metab. 21 (12), 2684–2693. 10.1111/dom.13858 31423699

[B64] Yan P. K. Zhang L. N. Feng Y. Qu H. Qin L. Zhang L. S. (2014). SHR3824, a novel selective inhibitor of renal sodium glucose cotransporter 2, exhibits antidiabetic efficacy in rodent models. Acta Pharmacol. Sin. 35 (5), 613–624. 10.1038/aps.2013.196 24786232 PMC4814034

[B65] Yu L. X. Amidon G. L. (1999). A compartmental absorption and transit model for estimating oral drug absorption. Int. J. Pharm. 186 (2), 119–125. 10.1016/s0378-5173(99)00147-7 10486429

[B66] Zambrowicz B. Freiman J. Brown P. M. Frazier K. S. Turnage A. Bronner J. (2012). LX4211, a dual SGLT1/SGLT2 inhibitor, improved glycemic control in patients with type 2 diabetes in a randomized, placebo-controlled trial. Clin. Pharmacol. Ther. 92 (2), 158–169. 10.1038/clpt.2012.58 22739142 PMC3400893

[B67] Zhang Y. F. Liu Y. M. Yu C. Wang Y. T. Zhan Y. Liu H. Y. (2021). Tolerability, pharmacokinetic, and pharmacodynamic profiles of henagliflozin, a novel selective inhibitor of sodium-glucose cotransporter 2, in healthy subjects following single- and multiple-dose administration. Clin. Ther. 43 (2), 396–409. 10.1016/j.clinthera.2020.12.012 33454124

[B68] Zhou F. Du N. Zhou L. Wang C. Ren H. Sun Q. (2022). The safety of sotagliflozin in the therapy of diabetes mellitus type 1 and type 2: A meta-analysis of randomized trials. Front. Endocrinol. (Lausanne) 13, 968478. 10.3389/fendo.2022.968478 36225203 PMC9548998

